# Assessing Public Knowledge and Practices Toward Bacterial Food Poisoning Prevention and Treatment Related to Meat Consumption in Saudi Arabia

**DOI:** 10.3390/healthcare14203376

**Published:** 2026-10-10

**Authors:** Nahed N. Mahrous, Samah Y. Labban, Ohoud S. Alhumaidan, Hibah Alharbi, Lina Abdullah, Farah Alfayez, Rawa Altaweel, Lama Alruhaimi, Bushra Salem Alsalem, Yahya F. Jamous

**Affiliations:** 1 Department of Biology, College of Science, University of Hafr Al-Batin, Hafar Al-Batin 39524, Saudi Arabia; 2 Department of Physiology, Faculty of Medicine, Umm Al-Qura University, Makkah 21955, Saudi Arabia; 3 Department of Clinical Laboratory Sciences, College of Applied Medical Sciences, King Saud University, Riyadh 12372, Saudi Arabia; 4 Saudi Food and Drug Authority, Riyadh 12843, Saudi Arabia; 5 Department of Pharmacy, College of Pharmacy, Princess Nourah Bint Abdulrahman University, Riyadh 11671, Saudi Arabia443000816@pnu.edu.sa (L.A.); 6 Alfa Food Analysis Laboratory, Food Sector, Riyadh 13226, Saudi Arabia; 7 Wellness and Preventive Medicine Institute, Health Sector, King Abdulaziz City for Science and Technology (KACST), Riyadh 11442, Saudi Arabia

**Keywords:** bacterial food poisoning, meat safety, food-handling practices, food-safety knowledge, food thermometer, Saudi Arabia

## Abstract

**Background:** Meat-related bacterial food poisoning remains an important public-health concern, and safe household food handling is essential for prevention. **Objective:** To assess food-poisoning knowledge, household meat-handling practices, and associated factors among adults in Saudi Arabia. **Methods:** A cross-sectional online survey using convenience sampling through social media was conducted from July 2025 to January 2026. Recognition scores were analyzed using ordinary least squares with HC3 robust covariance. Additional analyses were specified before model fitting but were not prospectively prespecified in the original protocol. The study-specific questionnaire was not formally validated. **Results:** Among 598 participants, improper meat storage (83.9%) and undercooked or raw meat consumption (83.3%) were the most frequently recognized causes. Only 10.9% reported using a food thermometer, while social media was the most frequently reported source of food-safety information. **Conclusions:** Important gaps remain in meat-related food-safety knowledge and practices in Saudi Arabia. Targeted education on safe storage, thawing, thermometer use, and appropriate responses to suspected foodborne illness may help improve household food-safety practices.

## 1. Introduction

Foodborne diseases remain a major global food-safety and public-health challenge. The World Health Organization (WHO) estimated that approximately 600 million people become ill and 420,000 die each year as a result of consuming contaminated food [1]. Foodborne illness may result from contamination with pathogenic bacteria, viruses, parasites, or other hazards. Major bacterial pathogens of concern include *Salmonella*, *Campylobacter*, *Listeria monocytogenes*, and Shiga toxin-producing *Escherichia coli* [2]. Food contamination may occur at multiple stages of the food chain, including processing, handling, storage, preparation, and consumption, emphasizing the need for effective food-safety practices throughout the farm-to-consumer continuum [3].

At the consumer level, food-safety knowledge and household food-handling behaviors are important components of foodborne disease prevention. Consumer studies have demonstrated that knowledge may be relatively good for basic hygiene practices while remaining inadequate for technically important areas such as temperature control [4]. Similar findings have been reported among university students in Saudi Arabia, who demonstrated relatively good knowledge of personal hygiene, cleanliness, and cross-contamination, but poorer knowledge and practices related to temperature control [5]. Food handlers working in Saudi university restaurants have likewise shown generally satisfactory food-safety knowledge and practices, although the findings emphasized the continuing importance of education and training, particularly in areas related to hygiene and time–temperature control [6]. These findings indicate that favorable overall food-safety knowledge may coexist with specific deficiencies in behaviors directly relevant to preventing foodborne illness.

Household meat preparation is particularly important because consumers may be exposed to foodborne pathogens through inappropriate handling and inadequate cooking. Direct observation of consumers preparing hamburgers at home identified frequent opportunities for cross-contamination, while only 4% of households used a meat thermometer to assess doneness [7]. A national Canadian survey similarly found that only 29% of participants reported using a food thermometer when cooking meat, despite high awareness of the foodborne illness risks associated with chicken and hamburgers [8]. More recent Canadian evidence showed that visual inspection remained the most frequently reported method for assessing meat doneness, supporting continued emphasis on proper storage, safe handling of raw meat and poultry, and food-thermometer use in consumer education [9]. Research specifically examining barriers to thermometer use has also shown that consumers commonly rely on alternative indicators of doneness or simply do not consider using a thermometer [10]. Together, these findings demonstrate an important distinction between general awareness of foodborne hazards and consistent use of objective food-safety practices.

Several studies have evaluated food-safety knowledge and practices in Saudi Arabia. Ayad et al. assessed household food-safety knowledge and practices and found that gender, age, and educational level influenced food-safety knowledge among Saudi respondents [11]. Among restaurant food handlers in Jeddah, generally high food-safety knowledge, attitude, and practice scores were reported; however, specific deficiencies were identified in practices related to cross-contamination, handwashing, thawing, leftover food, and thermometer use [12]. More recently, a nationwide study that was conducted following a foodborne botulism outbreak assessed food-poisoning knowledge, risk perception, and food-safety practices among 3779 participants and identified several gaps in consumer knowledge and behavior [13]. Another national study involving 1920 participants demonstrated insufficient food-related knowledge and considerable public interest in receiving additional information regarding food poisoning and food safety [14]. Furthermore, a Saudi study examining public interest, risk perception, and awareness of food safety found substantial gaps in understanding of the national food-safety system and highlighted the importance of the information sources used by consumers [15]. Collectively, these studies demonstrate continuing opportunities to strengthen consumer-focused food-safety education in Saudi Arabia.

Previous evidence indicates that food-safety knowledge does not consistently translate into appropriate practices, highlighting the importance of assessing both knowledge and actual household behaviors [16,17].

Food-safety education should address both knowledge and the practical implementation of recommended behaviors, as training and access to practical tools may improve food-safety practices and consumer-level interventions may help reduce foodborne risks within meat supply chains [18,19,20,21].

Social media is increasingly used to obtain and share health and food-safety information [22,23], and in Saudi Arabia it has been identified as an important source of food-related information that may support more targeted awareness strategies [14,15].

International evidence also supports assessing both food-safety knowledge and practical behavior, as consumer characteristics and unsafe handling practices have been associated with food-safety outcomes [24,25].

Despite the growing body of Saudi research on general food-safety knowledge, food-poisoning awareness, risk perception, and food-handling practices [5,6,11,12,13,14,15], evidence specifically integrating knowledge of bacterial food poisoning associated with meat consumption with household meat-handling practices remains limited. Existing Saudi studies have generally examined broad food-safety knowledge, specific population groups, or professional food handlers rather than simultaneously assessing bacterial food-poisoning knowledge, treatment-related perceptions, meat storage and thawing practices, cooking and reheating behaviors, methods used to determine meat doneness, perceived barriers to recommended practices, information sources, and sociodemographic and behavioral factors associated with knowledge within a single meat-focused consumer framework. Examining these dimensions together may provide a more comprehensive understanding of the knowledge–practice gap and identify specific behaviors that can be targeted through consumer food-safety interventions. Compared with previous Saudi studies that assessed general food-safety knowledge, food-poisoning awareness, or broader food-safety practices [11,13,14], the present study focuses specifically on meat-related bacterial food poisoning and integrates treatment-related perceptions, meat handling and storage, thawing, cooking and reheating behaviors, methods used to assess doneness, perceived barriers, information sources, and factors associated with knowledge within a single consumer-focused framework.

The primary objective of this study was to assess knowledge of bacterial food poisoning associated with meat consumption among adults residing in Saudi Arabia. Secondary objectives were to evaluate household meat-handling, storage, thawing, cooking, and reheating practices; examine treatment-related perceptions and responses to suspected meat-related food poisoning; identify perceived barriers to recommended meat-safety practices and major sources of food-safety information; and determine sociodemographic and behavioral factors associated with knowledge. By integrating these domains within a meat-specific framework, the study seeks to identify actionable knowledge and practice gaps that may inform targeted food-safety interventions.

## 2. Materials and Methods

### 2.1. Ethical Considerations

Ethical approval was obtained from the Institutional Review Board (IRB) of the Research Ethics Committee at King Abdulaziz City for Science and Technology (KACST), Saudi Arabia, before the commencement of data collection (approval No. 25006; on 16 March 2025). Participation was voluntary, and electronic informed consent was obtained from all participants before completing the questionnaire. Participants were informed about the purpose of the study and the confidentiality of their responses.

### 2.2. Study Design and Participants

A cross-sectional, survey-based study was conducted between July 2025 and January 2026 among adults residing in Saudi Arabia. Data were collected using a self-administered Arabic-language online questionnaire created and administered through Google Forms. The survey link was distributed using convenience sampling through social media platforms, including WhatsApp and Telegram, and through QR-code access at Princess Nourah bint Abdulrahman University. No population registry, sampling database, or other formal sampling frame was used. No stratified, quota-based, or probability sampling procedure was used, and recruitment was not proportionally stratified by geographic region; participant proportions across demographic and regional subgroups were therefore not predetermined. This non-probability recruitment approach may have contributed to demographic imbalance in the final sample, particularly the overrepresentation of females and younger adults, and therefore limits population-level representativeness. Because the survey link was distributed openly through social media, the total number of individuals who viewed or received the invitation could not be determined; therefore, a response rate could not be calculated. No financial or other compensation was offered for participation.

Individuals were eligible to participate if they were aged ≥18 years, resided in Saudi Arabia during the study period, and provided informed consent. Records with missing age were first excluded, followed by records with any missing value across the 30 questionnaire variables. Of the 624 records in the dataset, 598 complete records were retained for analysis and 26 were excluded.

### 2.3. Questionnaire and Study Measures

The questionnaire was developed with reference to previously published Saudi food-safety and food-poisoning surveys [11,13,14], which informed the selection of the main domains and topics assessed. Individual questionnaire items were not copied verbatim from a single validated instrument, and item-by-item source attribution was not available for all questions. The study-specific questionnaire was not formally validated through psychometric or content-validation procedures. Therefore, the resulting composite scores should be interpreted as study-specific measures rather than validated measures of objective knowledge.

The questionnaire collected information across five principal domains: (1) sociodemographic characteristics, including age, gender, educational level, occupation, and geographic region; (2) knowledge of bacterial food poisoning associated with meat consumption, including awareness of bacterial contamination, potential causes, symptoms, and treatment-related knowledge; (3) household meat-handling and storage practices; (4) thawing, cooking, reheating, and other food-safety behaviors; and (5) responses to suspected food poisoning, perceived barriers to recommended meat-safety practices, and sources of food-safety information. An English version of the questionnaire used in the study is provided in the Supplementary Material File S1.

Importantly, the survey did not ask participants to clinically identify or confirm the source of a previous food-poisoning episode. Rather, it assessed their knowledge of recognized risk factors and causes of meat-related bacterial food poisoning and their self-reported household meat-handling practices. No microbiological, clinical, or epidemiological confirmation of meat as the source of illness was performed.

Multiple-response questions were used for selected knowledge and behavioral domains in which participants could select more than one applicable response. Internal consistency was assessed using Cronbach’s alpha for both score definitions. Component frequencies, raw and corrected item–total correlations, and alpha after deleting each component were calculated. Corrected correlations excluded the corresponding component from the total. The questionnaire included factual recognition questions, self-reported awareness, reported practices, and beliefs. Questions 7 and 10 assessed self-reported awareness, whereas Question 11 asked about respondents’ usual actions when they or a family member developed symptoms. These questions therefore did not all measure objective knowledge in the same way. The questionnaire was not designed as a formal knowledge test with systematically constructed false distractor options. Instead, it included factual response options, self-reported awareness items, and “I do not know” responses where applicable.

Several household-practice questions were mandatory and did not include a “not applicable” option. Although these questions referred to respondents’ usual practices, the questionnaire did not distinguish personal behavior from observations of household members or hypothetical responses among participants without food-preparation responsibilities.

Meat consumption and direct responsibility for household food preparation were not eligibility criteria. Because some practice questions may have been less applicable to participants who did not consume or prepare meat, additional subgroup analyses were conducted among meat consumers and after excluding respondents who explicitly reported no responsibility for food preparation.

### 2.4. Sample Size Determination

The minimum required sample size was calculated using the single-population proportion approach. Assuming a population proportion of 50%, a 95% confidence level, and a 5% margin of error, the minimum required sample size was estimated to be 384 participants. Recruitment continued beyond the minimum required sample to improve the precision of the estimates and account for potentially incomplete or unusable responses. A total of 598 participants with eligible and sufficiently complete responses were included in the final analysis. The sample-size calculation was based on an estimation of a population proportion and was not specifically powered for the multivariable regression analyses. Accordingly, the multivariable analyses should be considered exploratory, with emphasis placed on effect estimates and confidence intervals rather than on formal hypothesis-testing power.

### 2.5. Study Outcomes and Knowledge Classification

The primary analytic outcome was a continuous nine-component recognition score assessing four causes and five symptoms of meat-related bacterial food poisoning. The original 14-component composite was retained only as a secondary sensitivity analysis. The original composite comprised six questions generating 14 scored components. The reduced score excluded perceived-awareness questions (Q7 and Q10), reported healthcare-seeking behavior (Q11), and both treatment components (Q13), whose interpretation required clinical context. Accordingly, Questions 7 and 10 were not treated as measures of objective knowledge in the primary recognition score.

The reduced score measures a narrower recognition domain and should not be considered independently validated. For the two single-response awareness questions (Questions 7 and 10), the highest self-reported awareness category received one point, and all other responses received zero points. These points reflected perceived awareness rather than objectively demonstrated knowledge. For multiple-response questions, each prespecified keyed option was scored independently as 1 if selected and 0 if not selected. Non-keyed options did not contribute points. When a respondent selected both a keyed option and ‘I do not know’ within the same question, the response was considered internally inconsistent. In sensitivity analyses, these cases were handled in two ways: (1) exclusion of respondents with inconsistent combinations, and (2) assignment of zero to all scored components of the affected question (Tables S1 and S2). For Question 11, selecting either consultation with a healthcare provider or attendance at a hospital contributed one point in total; selecting both did not provide an additional point.

The six knowledge questions generated a total of 14 scored responses. Correct responses included recognition of undercooked or raw meat consumption, cross-contamination, improper meat storage, and poor hand hygiene as potential causes; abdominal pain, fever, diarrhea, nausea, and vomiting as common symptoms; seeking professional medical care as an appropriate response to suspected food poisoning; and fluid replacement and physician-prescribed antibiotics as potential treatment options. The question assessing sources of food-safety information was excluded from the knowledge score.

A composite knowledge score was calculated by summing the individual scores, with a maximum attainable score of 14. The raw score was converted to a percentage using the formula (obtained score/14) × 100.

Secondary outcomes included household meat-handling, storage, thawing, cooking, and reheating practices; participants’ responses to suspected food poisoning; perceived barriers to adherence to recommended meat-safety practices; sources of food-safety information; and sociodemographic and behavioral factors associated with knowledge level.

### 2.6. Statistical Analysis

The original R analysis script was audited [26], and additional analyses were performed using Python 3.12.13, NumPy 2.3.5, pandas 2.2.3, SciPy 1.17.0, and scikit-learn 1.8.0. The analysis plan for these additional models was specified before model fitting; however, it was not prospectively established as part of the original study plan [27]. Categorical variables were summarized as frequencies and percentages. For multiple-response questions, each response option was coded and analyzed as a separate binary variable.

Associations between categorical variables and knowledge classification were initially evaluated using Pearson’s chi-square test or Fisher’s exact test, as appropriate. Both continuous scores were analyzed using ordinary least squares with HC3 heteroskedasticity-robust covariance and t-based inference. Adjusted differences were expressed in percentage points on a 0–100 scale. For secondary sensitivity analyses only, the original operational threshold of ≥10 points out of 14 was analyzed using Firth bias-reduced logistic regression. This threshold was not validated and was not used as the primary basis for interpretation.

Age group, sex, education, occupation, and region were retained in all adjusted models irrespective of statistical significance. Education below high school was combined with high school, and employed and self-employed respondents were combined on substantive grounds before model fitting. Each full-sample Firth model underwent internal validation using 500 case-bootstrap resamples with fixed model terms and coding. Apparent and optimism-corrected AUC, Brier score, and calibration slope were calculated (Table S3). Model selection was not based on the Hosmer–Lemeshow *p*-value [28]. No backward selection was used in the analyses. A demographic-only model was fitted, and each practice or belief from Questions 14–20 and 22–24 was examined separately with the same demographic adjustment set. Practices were not mutually adjusted or automatically treated as confounders of one another. Definitions of the analysis populations and revised variable categories are provided in Table S4.

Design-matrix rank and numerical convergence were checked. The Firth implementation was verified against an analytic two-by-two-table benchmark and independent numerical optimization. Continuous-score results are reported as adjusted mean differences with robust 95% confidence intervals. Firth logistic results are reported as odds ratios with approximate Wald 95% confidence intervals and matching two-sided Wald *p*-values. These intervals were not calculated using penalized profile likelihood.

## 3. Results

### 3.1. Participant Characteristics

A total of 598 participants were included in the final analysis. More than half of the participants were aged 18–24 years (52.5%), and females represented 75.4% of the study sample. Approximately half held a bachelor’s degree (51.3%), while 30.9% had completed high school. Students constituted the largest occupational group (47.5%), followed by employed participants (29.3%).

The Central Region accounted for the largest proportion of respondents (42.6%), followed by the Western (26.6%) and Eastern (19.2%) regions. Regarding meat consumption, 50.3% of participants described their consumption as modest, 36.1% as balanced, and 10.5% as high, while 3.0% reported that they did not consume meat (Table 1).

### 3.2. Knowledge of Bacterial Food Poisoning

Overall, 44.3% of participants reported being fully aware that meat-related food poisoning may result from bacterial contamination, while 38.0% had heard about this association but did not know the details. In contrast, 15.4% reported no awareness and 2.3% were unsure.

Knowledge of specific treatments was lower. Nearly half of the participants (47.0%) reported that they were unaware of specific treatments for meat-related bacterial food poisoning, while 28.9% indicated that they had only general knowledge.

Improper meat storage (83.9%) and consumption of undercooked or raw meat (83.3%) were the most frequently identified causes of bacterial food poisoning. Cross-contamination during meat preparation was identified by 61.5% of participants, and poor hand hygiene by 56.0%.

Abdominal pain was the most frequently recognized symptom (83.3%), followed by vomiting (71.9%), diarrhea (71.2%), and nausea (69.9%), whereas fever was identified by 42.3% of participants.

When asked what they usually do when they or a family member develops signs of food poisoning, 74.1% selected going to the hospital, 42.5% selected home remedies, and 33.4% selected consultation with a healthcare provider.

Regarding treatment options, 64.0% selected antibiotics prescribed by a physician, while 43.8% selected fluids or oral rehydration solutions (Table 2).

These descriptive item-level responses provide the distribution of knowledge-related answers, while inferential analyses examining factors associated with the knowledge outcomes are presented in Section 3.5.

### 3.3. Meat-Handling, Storage, and Cooking Practices

Most participants (72.9%) reported never consuming raw or undercooked meat. Hand hygiene was commonly reported, with 78.6% indicating that they always washed their hands before handling raw meat and 89.8% reporting that they always washed their hands afterward. The most frequently reported storage practice was keeping raw meat in the freezer until use (76.4%). More than half of the participants (58.5%) selected “always” when asked about using utensils specifically for handling raw and cooked meat. However, the question did not explicitly distinguish separate utensil sets for raw versus cooked meat.

Regarding beliefs about thawing and refreezing, 60.5% selected “only once” when asked how many cycles they considered safe. In a separate question about usual practices, 85.3% reported cooking meat immediately after thawing.

Bad or sour smell (83.8%) and unusual changes in color (76.3%) were the most commonly reported indicators that meat was unsafe for cooking.

To determine whether meat was thoroughly cooked, 53.2% reported cutting the meat to assess whether the juices ran clear, whereas 31.8% relied on smell or texture. Food-thermometer use was reported by 65/598 respondents overall (10.9%), 63/580 meat consumers (10.9%), 46/339 respondents remaining after excluding explicit non-responsibility for food preparation (13.6%), and 45/331 respondents in the intersection (13.6%).

More than half of the participants (54.5%) reported serving or consuming meat immediately after cooking. Reheating using a stove or oven was the most commonly reported method for leftover meat (52.0%), followed by reheating using a microwave (35.5%) (Table 3).

These descriptive practice frequencies are complemented by the adjusted analyses of associations between selected food-handling practices and knowledge outcomes presented in Section 3.5, and Supplementary Tables S6–S9.

### 3.4. Knowledge–Practice Gap and Suggested Strategies for Improvement

The most frequently reported barriers to following recommended meat-safety practices were not being responsible for food preparation at home (43.3%) and lack of knowledge or training (43.0%), followed by lack of time (29.3%) and the cost of appropriate tools or equipment (24.1%).

When asked whether they failed to follow recommended meat-safety practices despite being aware of them, 37.3% strongly disagreed and 24.6% disagreed, whereas 9.0% agreed and 10.7% strongly agreed.

Regarding the perceived effectiveness of spices in preventing bacterial contamination, 33.1% reported that they did not know or had never heard of using spices for this purpose. Most participants indicated that greater knowledge of meat safety would help improve their food-handling and cooking habits (84.4%), while 91.1% believed that following appropriate meat-safety practices could help prevent meat-related food poisoning.

Social media campaigns were the most frequently suggested strategy for improving meat-safety awareness (82.1%), followed by government health-awareness campaigns (61.9%) and school-based programs (55.5%) (Table 4).

Table 4 is retained to summarize participants’ perceived barriers and suggested strategies, while the main inferential analyses are presented separately in Section 3.5.

**Figure 1:**
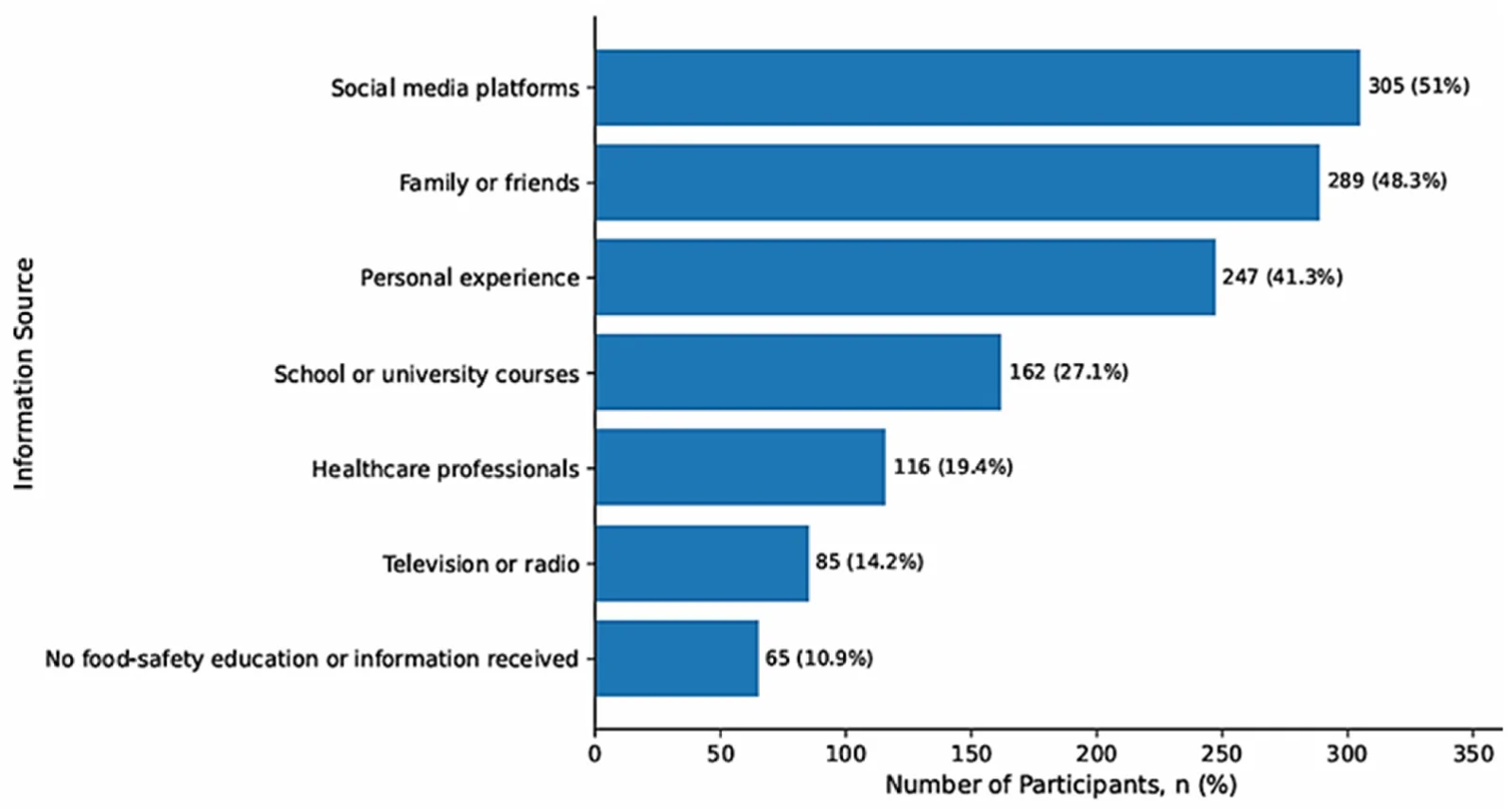
Frequencies and proportions of reported sources of meat-safety information.

Regarding sources of meat-safety information, social media platforms were the most frequently reported source (51.0%), followed by family or friends (48.3%) and personal experience (41.3%). School or university courses were reported by 27.1% of participants, healthcare professionals by 19.4%, and television or radio by 14.2%, while 10.9% reported receiving no food-safety education or information (Figure 1).

### 3.5. Factors Associated with Knowledge Outcomes Regarding Meat-Related Bacterial Food Poisoning

In the demographic-only model, female sex was associated with a 5.66-percentage-point higher reduced recognition score than male sex (95% CI: 0.18–11.14; *p* = 0.043). However, females represented 75.4% of the study sample, and this substantial sex imbalance may limit the generalizability and stability of sex-based comparisons. Accordingly, the observed association should be interpreted cautiously and in the context of the non-probability recruitment strategy. The employed/self-employed versus student contrast was imprecise after demographic adjustment: the reduced-score difference was 5.56 percentage points (95% CI: −4.90 to 16.01; *p* = 0.297).

In a separate demographically adjusted model, always washing hands after handling raw meat, compared with not always doing so, was associated with a 14.31-percentage-point higher reduced recognition score (95% CI: 6.05–22.56; *p* < 0.001). Freezer storage, compared with the combined refrigerated-storage categories, was associated with an 11.01-percentage-point higher reduced recognition score (95% CI: 5.16–16.86; *p* < 0.001; n = 595). This association does not establish that freezer storage is inherently safer.

Cooking thawed meat and subsequently freezing leftovers was associated with a lower reduced recognition score than cooking immediately (difference −11.17 percentage points; 95% CI: −18.56 to −3.78; *p* = 0.003). The association weakened among meat consumers who did not explicitly report non-responsibility for food preparation (difference −8.71; 95% CI: −18.72 to 1.30; *p* = 0.088). These findings do not establish that freezing cooked leftovers is unsafe (Table 5).

**Table 5:** Multivariable sensitivity analyses of knowledge score by demographic characteristics.

Contrast	Reduced 9: Difference (95% CI); *p*	Original 14: Difference (95% CI); *p*	Original Threshold: OR (95% CI); *p*
Age: 25–34 vs. 18–24	−7.23 (−17.56, 3.11); *p* = 0.170	−7.16 (−15.87, 1.56); *p* = 0.107	0.60 (0.30, 1.23); *p* = 0.166
Age: 35–44 vs. 18–24	−4.87 (−16.38, 6.64); *p* = 0.406	−5.98 (−15.37, 3.42); *p* = 0.212	0.75 (0.35, 1.65); *p* = 0.478
Age: 45–55 vs. 18–24	−5.57 (−17.52, 6.38); *p* = 0.360	−4.72 (−14.76, 5.32); *p* = 0.356	0.68 (0.30, 1.54); *p* = 0.360
Age: >55 vs. 18–24	−11.30 (−27.41, 4.81); *p* = 0.169	−9.83 (−23.48, 3.82); *p* = 0.158	0.40 (0.14, 1.18); *p* = 0.097
Sex: Female vs. Male	5.66 (0.18, 11.14); *p* = 0.043	4.96 (0.42, 9.49); *p* = 0.032	1.76 (1.15, 2.69); *p* = 0.010
Education: Diploma vs. High school or less	−3.22 (−13.59, 7.14); *p* = 0.541	−1.40 (−9.90, 7.09); *p* = 0.746	0.90 (0.41, 1.97); *p* = 0.784
Education: Bachelor vs. High school or less	2.49 (−2.98, 7.95); *p* = 0.372	3.58 (−0.88, 8.03); *p* = 0.116	1.26 (0.83, 1.93); *p* = 0.281
Education: Postgraduate vs. High school or less	8.00 (−2.03, 18.03); *p* = 0.118	11.36 (3.17, 19.54); *p* = 0.007	2.12 (1.04, 4.35); *p* = 0.040
Occupation: Employed/self-employed vs. Student	5.56 (−4.90, 16.01); *p* = 0.297	5.35 (−3.38, 14.08); *p* = 0.229	1.92 (0.94, 3.90); *p* = 0.073
Occupation: Unemployed vs. Student	4.21 (−6.04, 14.45); *p* = 0.420	4.70 (−3.92, 13.31); *p* = 0.285	1.48 (0.73, 3.00); *p* = 0.282
Occupation: Retired vs. Student	7.30 (−9.86, 24.46); *p* = 0.404	5.62 (−9.29, 20.53); *p* = 0.459	2.03 (0.66, 6.28); *p* = 0.220
Region: Central vs. Eastern	4.38 (−1.80, 10.56); *p* = 0.165	2.89 (−2.09, 7.86); *p* = 0.255	1.48 (0.92, 2.38); *p* = 0.110
Region: Western vs. Eastern	7.66 (1.32, 14.00); *p* = 0.018	6.25 (1.10, 11.41); *p* = 0.018	1.62 (0.97, 2.69); *p* = 0.063
Region: Northern vs. Eastern	−6.73 (−16.59, 3.12); *p* = 0.180	−6.24 (−14.19, 1.71); *p* = 0.124	0.51 (0.21, 1.21); *p* = 0.127
Region: Southern vs. Eastern	−0.30 (−11.65, 11.05); *p* = 0.958	1.63 (−8.15, 11.41); *p* = 0.744	0.92 (0.38, 2.25); *p* = 0.859

All demographic variables were retained together. Continuous estimates are adjusted differences in percentage points on a 0–100 scale, with HC3 robust 95% confidence intervals and t-based *p*-values. Binary estimates are Firth odds ratios (ORs) with approximate Wald intervals and matching Wald *p*-values. Reference categories appear in each contrast. No multiplicity adjustment. Associations between food-related practices and knowledge outcomes are presented in Table 6.

**Table 6:** Associations between food-related practices and knowledge outcomes in sensitivity analyses.

Contrast	N	Reduced 9: Difference (95% CI); *p*	Original 14: Difference (95% CI); *p*	Original Threshold: OR (95% CI); *p*
Q14: Ever vs. Never	598	−3.99 (−8.92, 0.93); *p* = 0.112	−2.71 (−6.84, 1.41); *p* = 0.197	0.81 (0.55, 1.20); *p* = 0.292
Q15: Always vs. Not always	598	5.43 (−0.31, 11.16); *p* = 0.064	7.89 (3.05, 12.74); *p* = 0.001	1.46 (0.96, 2.21); *p* = 0.076
Q16: Always vs. Not always	598	14.31 (6.05, 22.56); *p* < 0.001	13.81 (6.94, 20.67); *p* < 0.001	2.71 (1.47, 4.99); *p* = 0.001
Q17: Freezer vs. Refrigerator	595	11.01 (5.16, 16.86); *p* < 0.001	7.84 (2.99, 12.68); *p* = 0.002	2.33 (1.47, 3.70); *p* < 0.001
Q17: Room temperature vs. Refrigerator	595	−5.36 (−24.67, 13.95); *p* = 0.586	−6.46 (−23.97, 11.06); *p* = 0.469	1.02 (0.29, 3.62); *p* = 0.978
Q18: Always vs. Less than always	598	−0.68 (−4.91, 3.55); *p* = 0.753	2.11 (−1.38, 5.60); *p* = 0.235	1.21 (0.86, 1.70); *p* = 0.282
Q19: Twice/multiple vs. Only once	598	−9.24 (−15.56, −2.92); *p* = 0.004	−9.08 (−14.40, −3.77); *p* < 0.001	0.53 (0.32, 0.86); *p* = 0.010
Q19: Unsure vs. Only once	598	−5.29 (−10.66, 0.08); *p* = 0.053	−7.09 (−11.48, −2.70); *p* = 0.002	0.56 (0.37, 0.85); *p* = 0.007
Q20: Cook then freeze leftovers vs. Cook immediately	598	−11.17 (−18.56, −3.78); *p* = 0.003	−11.20 (−17.61, −4.80); *p* < 0.001	0.26 (0.13, 0.54); *p* < 0.001
Q20: Refreeze uncooked vs. Cook immediately	598	−9.65 (−20.87, 1.57); *p* = 0.092	−9.87 (−19.43, −0.30); *p* = 0.043	0.50 (0.23, 1.07); *p* = 0.074
Q22: Smell/texture vs. Juices	598	−4.62 (−9.25, 0.01); *p* = 0.051	−4.30 (−8.11, −0.48); *p* = 0.027	0.78 (0.53, 1.14); *p* = 0.195
Q22: Thermometer vs. Juices	598	−11.52 (−19.55, −3.49); *p* = 0.005	−4.56 (−11.15, 2.03); *p* = 0.175	1.07 (0.61, 1.88); *p* = 0.825
Q22: No check vs. Juices	598	−19.40 (−34.27, −4.52); *p* = 0.011	−15.62 (−28.03, −3.21); *p* = 0.014	0.41 (0.16, 1.03); *p* = 0.059
Q23: After 30 min vs. Within 30 min	598	−6.72 (−12.80, −0.64); *p* = 0.030	−6.82 (−11.92, −1.71); *p* = 0.009	0.71 (0.45, 1.10); *p* = 0.123
Q24: Microwave vs. Stove/oven	598	−4.94 (−9.45, −0.44); *p* = 0.032	−2.07 (−5.73, 1.59); *p* = 0.268	0.79 (0.55, 1.15); *p* = 0.216
Q24: No reheating/cold vs. Stove/oven	598	−10.89 (−18.48, −3.30); *p* = 0.005	−6.70 (−13.13, −0.27); *p* = 0.041	0.56 (0.32, 0.98); *p* = 0.043

Q14, raw/undercooked meat consumption; Q15/Q16, handwashing before/after raw meat; Q17, storage; Q18, utensil use (separate sets not established); Q19, belief about safe thaw/refreeze cycles; Q20, action after thawing; Q22, assessment of doneness; Q23, serving time; Q24, reheating. N = 595 for storage models after excluding three other/no-storage responses; otherwise, N = 598. CI, confidence interval; OR, odds ratio. Continuous estimates use HC3 robust inference; binary estimates use approximate Wald inference. The association between food-thermometer use and knowledge differed across outcome definitions. Thermometer use was associated with a lower reduced recognition score, whereas no significant association was observed with the original 14-component score or the operational 70% threshold, indicating that this finding was not consistent across analytic definitions.

### 3.6. Knowledge Score Distribution and Internal Consistency

**Figure 2:**
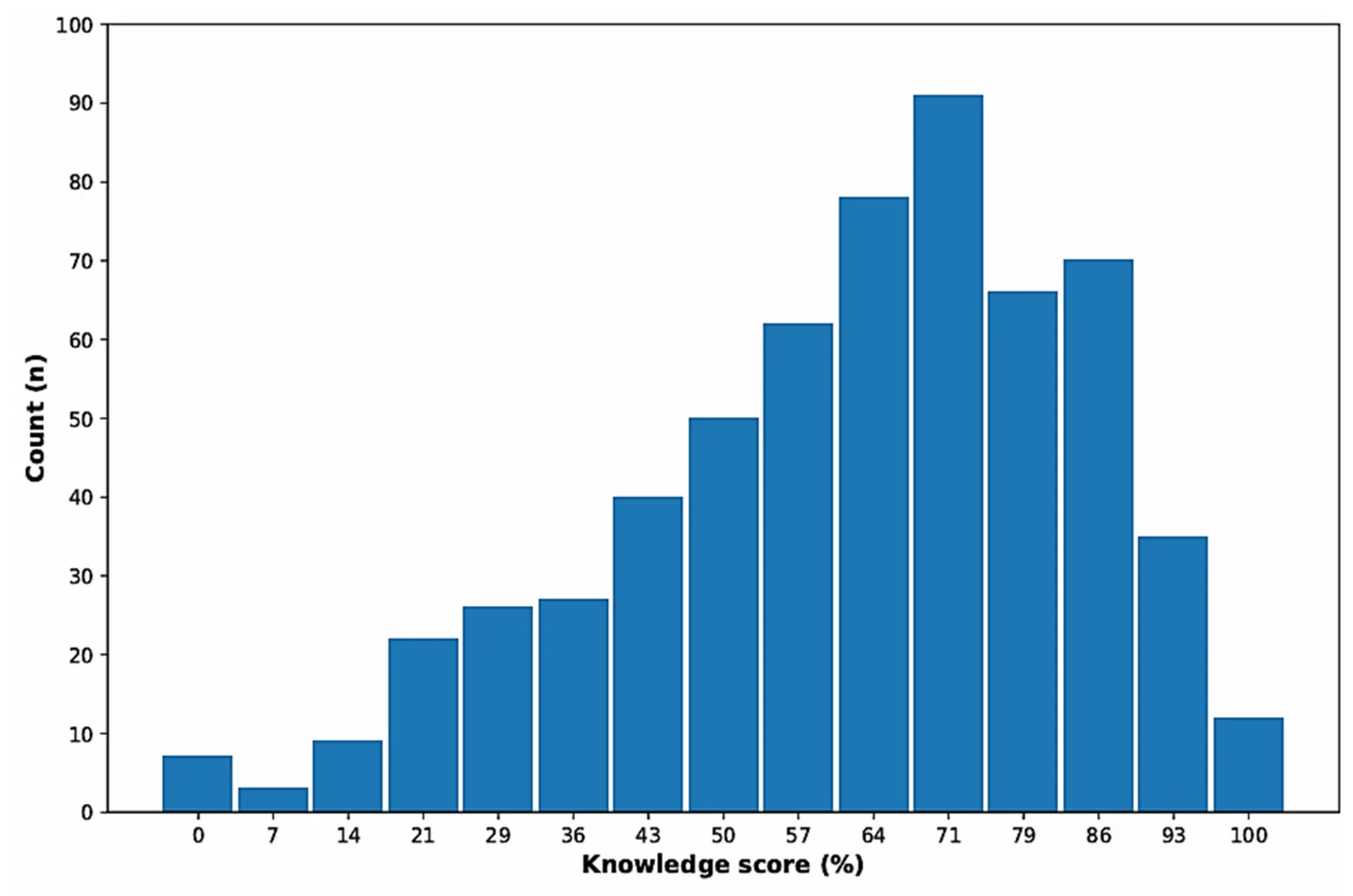
Frequency distribution of the knowledge score. The mean ± SD was 62.27 ± 21.67, and the median (IQR) was 64.29 (50.00–78.57). The distribution deviated significantly from normality (Shapiro–Wilk test, *p* < 0.001; n = 598).

The reduced recognition score had a mean ± SD of 6.23 ± 2.35 out of 9 and a median of 7 (IQR: 5–8). The original composite retained a mean of 62.27 ± 21.67 on the 0–100 scale and a median of 64.29 (IQR: 50.00–78.57). The distribution of knowledge scores is presented in Figure 2.

Cronbach’s alpha was 0.754 for the original 14-component composite and 0.766 for the reduced nine-component recognition score. Corrected item–total correlations for the original composite ranged from 0.166 to 0.535, and alpha after deleting an individual component ranged from 0.722 to 0.759. This internal-consistency estimate does not establish content or criterion validity.

## 4. Discussion

The present findings indicate a clear knowledge–practice gap in meat-related food safety. While participants generally recognized common causes and symptoms, important deficiencies remained in treatment-related knowledge and objective assessment of meat doneness, particularly food-thermometer use. These findings are broadly consistent with previous Saudi studies reporting reasonable general food-safety awareness alongside specific gaps in knowledge and household practices [11,13,14,15]. Accordingly, the findings should be interpreted as reflecting participants’ knowledge and perceptions of meat-related food-poisoning risks rather than confirmed attribution of individual food-poisoning episodes to meat consumption or improper meat handling.

Participants generally showed good recognition of major causes and symptoms of bacterial food poisoning. This pattern is broadly consistent with previous Saudi studies showing that consumers may demonstrate reasonable awareness of general food-safety concepts while retaining important gaps in more specific areas of knowledge and practice [11,13,14,15]. In the nationwide Saudi study conducted following a foodborne botulism outbreak, Alhuzaimi et al. similarly found moderate food-poisoning knowledge alongside suboptimal food-safety practices [13]. More recent Saudi research among food-truck consumers also found that women demonstrated higher food-safety knowledge and foodborne-pathogen awareness than men, reinforcing the presence of demographic differences in consumer food-safety knowledge [29].

Treatment-related knowledge represented a distinct area of uncertainty. Nearly half of participants reported no knowledge of specific treatments for meat-related bacterial food poisoning, while 64.0% selected physician-prescribed antibiotics as a possible treatment option. Because the survey specifically referred to antibiotics prescribed by a physician, this finding should not be interpreted as evidence of inappropriate antimicrobial use. Rather, it may reflect uncertainty regarding the circumstances in which supportive care, medical evaluation, or antimicrobial therapy may be appropriate. From a food-safety communication perspective, educational messages could therefore extend beyond prevention and include clear guidance on appropriate responses to suspected foodborne illness and when professional medical assessment should be sought.

The findings related to household meat preparation are particularly important. Although most participants reported frequent handwashing, freezer storage, and avoidance of raw or undercooked meat, the methods used to determine meat doneness were less consistent with objective food-safety practices. More than half of respondents reported cutting meat to determine whether the juices ran clear, 31.8% relied on smell or texture, and only 10.9% reported using a food thermometer. This pattern is consistent with previous consumer studies documenting reliance on visual or sensory indicators rather than temperature measurements [7,8,9,10]. Foodbook 2.0 likewise identified unsafe practices related to checking meat and poultry doneness and found that visual inspection remained common among consumers [9].

The low level of thermometer use observed in the present study is particularly notable because it is consistent with previous Saudi evidence. Alhuzaimi et al. reported routine thermometer use during cooking in only 2.7% of their Saudi sample [13], while Javed and Al-Mohaithef identified limitations in thermometer availability and temperature monitoring even within restaurant settings in Dammam [30]. These findings suggest that limited familiarity with food thermometers may extend across both household and professional food-preparation contexts in Saudi Arabia. Internationally, barriers to food-thermometer use include lack of habit, limited perceived necessity, and reliance on alternative indicators of doneness [10]. Importantly, behavioral interventions can improve thermometer adoption. Takeuchi et al. demonstrated a substantial increase in consumer readiness and actual thermometer use following a targeted educational intervention [31]. This suggests that campaigns focused on practical demonstrations rather than information alone may be particularly relevant to the Saudi context.

The responses suggested a possible discrepancy between perceived awareness and reported food-safety practices; however, actual behavior was not observed, and personal involvement in food preparation was not directly established. Although 91.1% of participants believed that following meat-safety practices could prevent food poisoning and 84.4% believed that additional knowledge would improve their practices, several barriers were reported. Lack of knowledge or training, limited responsibility for food preparation within the household, time constraints, and the cost of appropriate equipment were among the most frequently identified obstacles. Previous evidence from Gulf countries has similarly demonstrated that food-safety knowledge does not consistently translate into safe practice [16]. More broadly, recent reviews of consumer food-safety research have highlighted persistent differences between knowledge, attitudes, and observed or self-reported practices across demographic and cultural settings [17,32,33].

This knowledge–practice gap has important implications for intervention design. Educational approaches that simply provide information may improve knowledge without necessarily producing equivalent changes in behavior. Yeargin et al. emphasized the importance of moving beyond passive knowledge transfer toward strategies that facilitate practical adoption and implementation [18]. Madjdian et al. further demonstrated that combining training with practical tools can improve food-safety behaviors [19]. Similarly, a recent systematic review and meta-analysis published in *Foods* found that online food-safety education produced measurable but variable improvements in knowledge, attitudes, and practices, with barriers including limited attention, time constraints, and difficulty engaging with educational material [34]. These findings indicate that consumer education should be behavior-oriented, concise, practical, and tailored to specific barriers rather than relying exclusively on general awareness messages.

The present findings are also consistent with emerging evidence emphasizing the importance of household food safety as a distinct area of research. A recent scoping review of home food-safety knowledge, attitudes, and practices identified substantial methodological and behavioral variability across consumer studies and emphasized the importance of studying food handling within domestic settings [32]. Similarly, a 2026 *Foods* review of urban consumers found that food-safety knowledge and positive attitudes frequently coexisted with unsafe practices and that sociodemographic characteristics such as gender, education, employment, and income were associated with variation in food-safety behaviors [33]. The present study adds to this literature by focusing specifically on bacterial food poisoning associated with meat and by integrating knowledge, meat-handling practices, perceived barriers, treatment-related perceptions, and information sources within the same population.

Social media emerged as a particularly important component of food-safety communication. It was the most frequently reported source of meat-safety information and was also the most commonly suggested channel for future awareness campaigns. This observation is consistent with Saudi evidence showing substantial preference for digital communication and short visual formats for food-related awareness messages [14,15]. Social media offers considerable advantages in terms of reach, immediacy, and accessibility; however, these benefits are accompanied by challenges regarding credibility, accuracy, and consistency of information [22,23]. A systematic review specifically examining social media for food-safety risk communication highlighted both its broad reach and the importance of trust and evidence-based messaging [35]. More recent research has similarly emphasized the potential value of expert participation and scientifically grounded communication in online discussions concerning food-related risks [36].

Social media may therefore represent an effective channel for Saudi food-safety interventions if it is used strategically. Rather than delivering broad messages such as “cook meat thoroughly,” digital interventions could demonstrate specific behaviors, including correct food-thermometer placement, safe thawing, the separation of raw and cooked foods, appropriate refrigerator and freezer storage, and safe reheating. Evidence from food-safety risk-communication research indicates that perceived severity, vulnerability, self-efficacy, and information needs can influence consumers’ engagement with food-safety information on social media [37]. Thus, communication strategies that combine clear risk information with practical actions may be more likely to influence behavior than awareness messaging alone.

The multivariable analyses identified several sociodemographic and behavioral factors associated with the reduced recognition score. Therefore, the association with female sex should be interpreted as an adjusted association within this sample rather than as evidence of strong predictive performance of the demographic model. This finding is consistent with previous Saudi research in which gender differences were observed in household food-safety knowledge [11], and with recent Saudi research among food-truck customers showing significantly higher food-safety knowledge and pathogen awareness among women [29]. International evidence also demonstrates gender-related variation in consumer food-safety knowledge and practices [9,33]. These differences may reflect variation in household food-preparation responsibilities, exposure to food-safety information, or previous experience with food handling; however, these explanations were not directly measured in the present study and should therefore be considered hypotheses rather than established mechanisms.

Employment status was not significantly associated with the reduced recognition score after demographic adjustment; the employed/self-employed versus student contrast was 5.56 percentage points (95% CI: −4.90 to 16.01; *p* = 0.297). Previous studies have identified occupation, educational exposure, and socioeconomic factors as potential determinants of food-safety knowledge and behavior [16,17,33]. The present sample contained a relatively high proportion of students, which may partly explain the observed association. Nevertheless, the cross-sectional design does not permit determination of whether employment itself improves food-safety knowledge or whether the association reflects differences in age, experience, responsibility for food preparation, or other unmeasured factors.

Behavioral factors were also associated with knowledge. Participants who reported always washing their hands after handling raw meat had a 14.31-percentage-point higher reduced recognition score than those who did not always do so (95% CI: 6.05–22.56; *p* < 0.001). This association should be interpreted as a relationship between reported hygiene practice and recognition-based knowledge rather than as evidence of causality.

Reported freezer storage was associated with an 11.01-percentage-point higher reduced recognition score compared with refrigerated storage (95% CI: 5.16–16.86; *p* < 0.001). This association does not establish that freezer storage is inherently safer than refrigerated storage because storage duration and temperature were not assessed.

Conversely, participants who reported cooking thawed meat and subsequently freezing leftovers had an 11.17-percentage-point lower reduced recognition score than those who cooked meat immediately after thawing (95% CI: −18.56 to −3.78; *p* = 0.003). The observed association with freezing cooked leftovers should not be interpreted as evidence that this practice is inherently unsafe.

These findings support the broader observation that knowledge and household food-handling practices are related. However, the direction of this relationship cannot be determined from cross-sectional data. Greater knowledge may encourage safer practices, while individuals who more frequently prepare, store, and handle food may acquire greater food-safety knowledge through experience. Similar associations between food-safety knowledge and practices have been observed among consumers and food handlers in Saudi Arabia and other settings [24,25,38].

The regional and demographic differences observed in the bivariate analyses also warrant consideration. These comparisons were unadjusted and should be interpreted cautiously. Such variation may reflect differences in educational exposure, urbanization, information access, or sample composition. However, because recruitment was performed online and was not proportionally stratified by region, these regional comparisons should be interpreted cautiously. The study among Hajj pilgrims also demonstrated associations between demographic characteristics and food-safety knowledge and practices, illustrating the heterogeneity of food-safety behavior among populations encountered within Saudi Arabia [38].

The study has several strengths. The final sample of 598 participants exceeded the calculated minimum sample size and included respondents from all major regions of Saudi Arabia. The questionnaire captured multiple dimensions relevant to consumer meat safety, including knowledge of bacterial food poisoning, handling and storage, thawing, cooking and reheating, treatment-related perceptions, perceived barriers, and information sources. The analyses used fixed demographic adjustment, alternative score definitions, subgroup sensitivity analyses, and Firth logistic estimation.

### Study Limitations

Several limitations should nevertheless be acknowledged. First, the cross-sectional design precludes causal inference and does not establish the temporal relationship between knowledge and food-handling behavior. Second, recruitment through social media represents a non-probability sampling approach and may have introduced selection bias toward individuals who were younger, more digitally connected, or more interested in health and food-safety topics. Females represented 75.4% of the sample and participants aged 18–24 years accounted for 52.5%, limiting the extent to which the findings can be generalized to the entire Saudi adult population. Third, all behavioral measures were self-reported and may therefore be subject to recall and social-desirability bias. Participants may report practices that they perceive as desirable even when these behaviors are not consistently implemented. Recent reviews of home food-safety studies have similarly emphasized methodological limitations associated with questionnaire-based assessment and heterogeneity in KAP measurement [32,33].

The original knowledge score combined factual recognition with self-reported awareness and reported healthcare-seeking behavior, which limits its interpretation as a measure of objective knowledge. Furthermore, the cause and symptom recognition items did not include systematically constructed false distractors; therefore, these items may reflect recognition rather than fully discriminating actual knowledge and may overestimate participants’ knowledge. In addition, several household-practice questions lacked a “not applicable” option. Food-preparation responsibility was captured only through a multiple-response barriers question; failure to select non-responsibility cannot confirm involvement in food preparation. Practice analyses were repeated among meat consumers (n = 580), after excluding respondents explicitly reporting non-responsibility for food preparation (n = 339), and in the intersection of these groups (n = 331). Original practice-response distributions across the applicability subgroups are presented in Table S5, and the corresponding adjusted practice/belief association analyses are provided in Tables S6–S9. Failure to select the non-responsibility option does not establish preparation involvement; these groups should not be described as confirmed household food preparers.

Fourth, several response categories contained small numbers of participants, producing wide confidence intervals for some regression estimates. Finally, the study assessed reported knowledge and behavior but did not directly observe household food preparation or perform microbiological assessment of meat products.

From a practical perspective, the findings suggest that food-safety interventions in Saudi Arabia should prioritize a limited number of clearly actionable household behaviors. Food-thermometer use represents an obvious target, given its very low prevalence in this study and previous Saudi research [13,30]. Safe thawing, appropriate storage, prevention of cross-contamination, and evidence-based responses to suspected food poisoning should also be emphasized. Intervention studies demonstrate that structured educational packages and repeated practical reinforcement can improve food-handling practices [39], while online programs can provide scalable methods for delivering food-safety education [34]. Given the strong preference for social media identified in the present study, short videos, demonstrations, infographics, and messages distributed through trusted Saudi health and food-safety authorities may provide particularly appropriate delivery formats.

Recent consumer research in Saudi Arabia also indicates continuing demand for targeted food-safety education. The 2026 study among food-truck customers demonstrated measurable gaps in foodborne-pathogen awareness and identified demographic differences in knowledge [29], while recent international consumer research has emphasized the influence of lifestyle, perceived risk, and consumer characteristics on food-safety perceptions [40]. These findings support moving toward segmented rather than uniform food-safety communication, with messages tailored to specific audiences, behaviors, and information needs.

Overall, the present study demonstrates that awareness of meat-associated bacterial food poisoning among surveyed adults in Saudi Arabia coexists with important gaps in detailed knowledge and household food-handling practices. The findings suggest that future strategies should move beyond general awareness toward practical, behavior-focused interventions that address specific deficiencies, particularly objective assessment of meat doneness, safe storage and thawing, and evidence-based food handling. Integrating reliable digital communication with practical food-safety tools and targeted education may provide a useful approach for narrowing the gap between knowledge and everyday behavior.

## 5. Conclusions

This study identified important gaps in knowledge and household practices related to meat-associated bacterial food poisoning among adults in Saudi Arabia. Although awareness of common causes and symptoms was generally high, treatment-related knowledge and food-thermometer use remained limited. The findings support targeted, practical food-safety education focusing on safe meat storage and thawing, the prevention of cross-contamination, objective assessment of meat doneness, and appropriate responses to suspected foodborne illness. Digital and social-media-based communication may provide useful channels for delivering these interventions.

Given the prominent role of social media as a source of meat-safety information, targeted and evidence-based digital communication may provide a practical approach for improving consumer food-safety knowledge and promoting safer household practices. Future interventions should prioritize practical behaviors, including appropriate meat storage and thawing, prevention of cross-contamination, and the use of food thermometers to assess doneness. Future studies using probability-based or otherwise more representative sampling methods and intervention-based or longitudinal designs are warranted to confirm these findings and evaluate the effectiveness of targeted food-safety strategies in Saudi Arabia.

## Figures and Tables

**Table 1:** Demographic characteristics of the study participants (n = 598).

Characteristic	Category	n (%)
Age (years)	18–24	314 (52.5)
	25–34	89 (14.9)
	35–44	85 (14.2)
	45–55	67 (11.2)
	>55	43 (7.2)
Gender	Male	147 (24.6)
	Female	451 (75.4)
Highest level of education	Less than high school	10 (1.7)
	High school	185 (30.9)
	Diploma/technical certificate	38 (6.4)
	Bachelor’s degree	307 (51.3)
	Postgraduate degree (Master’s/PhD)	58 (9.7)
Occupation	Student	284 (47.5)
	Employed	175 (29.3)
	Unemployed	76 (12.7)
	Self-employed	20 (3.3)
	Retired	43 (7.2)
Region of residence	Eastern Region	115 (19.2)
	Central Region	255 (42.6)
	Western Region	159 (26.6)
	Northern Region	40 (6.7)
	Southern Region	29 (4.8)
Meat consumption level	Does not consume meat (vegetarian)	18 (3.0)
	Modest consumption; approximately 25% of meals or less	301 (50.3)
	Moderate consumption; approximately 50% of meals	216 (36.1)
	High consumption; approximately 75% of meals or more	63 (10.5)

**Table 2:** Participants’ responses to knowledge items regarding bacterial food poisoning.

Characteristic	Description	n (%)
Awareness that meat-related food poisoning may result from bacterial contamination	Yes, I am fully aware of this.	265 (44.3)
	I have heard about it but do not know the details	227 (38.0)
	No, I was not aware of this	92 (15.4)
	I am not sure	14 (2.3)
Awareness of specific treatments for meat-related bacterial food poisoning	Yes, I am aware of specific treatments	86 (14.4)
	I have a general idea but not the details	173 (28.9)
	No, I am not aware of any specific treatments	281 (47.0)
	I am not sure	58 (9.7)
Causes of bacterial food poisoning *	Consuming undercooked or raw meat	498 (83.3)
	Cross-contamination during meat preparation	368 (61.5)
	Improper meat storage	502 (83.9)
	Poor hand hygiene	335 (56.0)
	Others	13 (2.2)
	I do not know	21 (3.5)
Symptoms commonly linked to bacterial food poisoning from contaminated meat *	Abdominal pain	498 (83.3)
	Fever	253 (42.3)
	Diarrhea	426 (71.2)
	Nausea	418 (69.9)
	Vomiting	430 (71.9)
	I do not know	45 (7.5)
Actions taken when the participant or a family member shows signs of meat poisoning *	Consult a healthcare provider	200 (33.4)
	Go to the hospital	443 (74.1)
	I do not know what to do	24 (4.0)
	Wait for symptoms to pass without intervention	71 (11.9)
	Take home remedies	254 (42.5)
	Self-medicate with over-the-counter medications	106 (17.7)
Potential treatment options for meat-related bacterial food poisoning *	Drinking fluids and oral rehydration solutions	262 (43.8)
	Home remedies	187 (31.3)
	Taking antibiotics prescribed by a doctor	383 (64.0)
	Using over-the-counter anti-diarrheal medications (e.g., loperamide)	138 (23.1)
	I do not know	92 (15.4)

* Multiple-response item.

**Table 3:** Meat-handling, storage, and cooking practices.

Characteristic	Description	n (%)
Frequency of raw meat consumption	Never—I do not consume raw or undercooked meat.	436 (72.9)
	Rarely—I have tried it once or a few times.	72 (12.0)
	Occasionally—I eat it from time to time.	72 (12.0)
	Frequently—I consume raw or undercooked meat regularly.	18 (3.0)
Frequency of washing hands before handling raw meat	Never—I was not aware it was necessary.	22 (3.7)
	Rarely—I do not usually think about it.	28 (4.7)
	Sometimes—I wash my hands, but not every time.	78 (13.0)
	Always—I wash my hands every time before handling raw meat.	470 (78.6)
Frequency of washing hands after handling raw meat	Never—I was not aware it was necessary.	15 (2.5)
	Rarely—I usually forget or skip this step.	16 (2.7)
	Sometimes—I wash my hands, but not every time.	30 (5.0)
	Always—I wash my hands immediately after handling raw meat.	537 (89.8)
Storage method of raw meat at home	In the refrigerator, separated from other foods.	93 (15.6)
	In the refrigerator, but stored together with other foods (not separated).	32 (5.4)
	In the freezer until ready to use.	457 (76.4)
	At room temperature (not refrigerated or frozen).	13 (2.2)
	Others	2 (0.3)
	I do not store raw meat at home.	1 (0.2)
Frequency of using certain utensils (e.g., knives, cutting boards) specifically for handling raw and cooked meat	Never	39 (6.5)
	Rarely	36 (6.0)
	Sometimes	65 (10.9)
	Frequently	108 (18.1)
	Always	350 (58.5)
Beliefs about the number of safe thawing and refreezing cycles	Only once	362 (60.5)
	Twice	70 (11.7)
	Multiple times, as many as needed	28 (4.7)
	I do not know/Not sure	138 (23.1)
After thawing meat, what do you usually do?	Cook it immediately after thawing	510 (85.3)
	Cook it and then freeze leftovers again.	52 (8.7)
	Refreeze the meat without cooking it	23 (3.8)
	I thaw and refreeze meat multiple times without cooking	13 (2.2)
How do you know if the meat is not safe for cooking?	Bad or sour smell	501 (83.8)
	Visible mold or slimy texture	417 (69.7)
	Unusual change in color	456 (76.3)
	I do not know	39 (6.5)
Methods used to check if meat is thoroughly cooked	Cutting the meat to see if juices run clear	318 (53.2)
	Relying on smell or texture	190 (31.8)
	Using a food thermometer to check internal temperature	65 (10.9)
	I do not check if meat is thoroughly cooked	25 (4.2)
After cooking meat, how soon do you usually serve or eat it?	Immediately	326 (54.5)
	Within 30 min	153 (25.6)
	Within 1 h	72 (12.0)
	After more than 1 h	47 (7.9)
How do you typically reheat leftover meat?	On the stove or in the oven	311 (52.0)
	Using a microwave	212 (35.5)
	I do not usually reheat leftover meat	58 (9.7)
	Eat it cold without reheating	17 (2.8)

**Table 4:** Knowledge–practice gap and participants’ suggestions for improvement.

Characteristic	Description	n (%)
Challenges preventing adherence to meat-safety practices *	Cost of proper tools or equipment (e.g., thermometer and cutting boards)	144 (24.1)
	Cultural or traditional beliefs	101 (16.9)
	Not responsible for food preparation at home	259 (43.3)
	Lack of knowledge or training	257 (43.0)
	Lack of time	175 (29.3)
	Food safety is not considered a priority	28 (4.7)
Despite knowing the correct practices, I do not always follow them in daily life	Strongly disagree	223 (37.3)
	Disagree	147 (24.6)
	Neutral	110 (18.4)
	Agree	54 (9.0)
	Strongly agree	64 (10.7)
Perceived effectiveness of spices in preserving meat from bacterial contamination *	I do not think they have a significant effect on preserving meat	170 (28.4)
	I think they are very effective and help reduce bacterial contamination	118 (19.7)
	Partially effective; other preservation methods are also needed	112 (18.7)
	I do not know/have never heard of using spices for this purpose	198 (33.1)
Having more knowledge about meat safety would help improve food-handling and cooking habits	Yes	505 (84.4)
	Maybe, it could help	60 (10.0)
	No	33 (5.5)
Following meat-safety practices can help prevent meat poisoning	Yes	545 (91.1)
	No	24 (4.0)
	Do not know	29 (4.8)
Best ways to raise awareness about meat safety in Saudi Arabia *	Community workshops or events	263 (44.0)
	Government health-awareness campaigns	370 (61.9)
	Mobile applications or SMS alerts	228 (38.1)
	Social media campaigns (e.g., Twitter, Instagram, Snapchat)	491 (82.1)
	Television and radio advertisements	250 (41.8)
	School-based programs	332 (55.5)
	Others	11 (1.8)

* Multiple-response item.

## Data Availability

The data presented in this study are available on request from the corresponding author. The data are not publicly available due to privacy and ethical restrictions.

## Supplementary Materials

The following supporting information can be downloaded at: https://www.mdpi.com/article/10.3390/healthcare14203376/s1, Table S1: Practice/belief associations: Exclude inconsistent responses; Table S2: Practice/belief associations: Contradictory question scored zero; Table S3: Bootstrap internal validation of full-sample Firth models; Table S4: Analysis populations and category definitions; Table S5: Original practice responses by applicability subgroup; Table S6: Practice/belief associations: Full; Table S7. Practice/belief associations: Meat consumers; Table S8: Practice/belief associations: Exclude explicit non-responsibility; Table S9: Practice/belief associations: Intersection. Supplementary File S1: Questionnaire English version of the questionnaire used in the study.
