# Supplementary material for: Assessing Public Knowledge and Practices Toward Bacterial Food Poisoning Prevention and Treatment Related to Meat Consumption in Saudi Arabia
Source: Healthcare (Basel). 2026 Oct 10;14(20):3376. doi: 10.3390/healthcare14203376 (PMC13704610; doi:10.3390/healthcare14203376)
Supplement: Supplementary file 1 [file healthcare-14-03376-s001.zip › healthcare-4548268-supplementary.pdf]

Table S1. Practice/belief associations: Exclude inconsistent responses

Each row comes from a separate question model adjusted for age, sex, education, occupation, and region. Entries give estimate (95% CI); p. All fitted contrasts are shown. Continuous-score estimates are percentage-point differences; binary estimates are ORs. Full demographic coefficients for every model are supplied in the analysis archive.

Contrast	N	Reduced 9 difference	Original 14 difference	Original threshold OR
Q14: Ever vs Never	571	-4.01 (-9.02, 1.01); p = 0.117	-2.74 (-6.95, 1.47); p = 0.202	0.85 (0.57, 1.27); p = 0.420
Q15: Always vs Not always	571	5.46 (-0.45, 11.37); p = 0.070	8.17 (3.19, 13.15); p = 0.001	1.46 (0.95, 2.24); p = 0.086
Q16: Always vs Not always	571	14.32 (5.90, 22.75); p ≤ 0.001	13.94 (6.96, 20.93); p ≤ 0.001	2.72 (1.45, 5.08); p = 0.002
Q17: Freezer vs Refrigerator	568	11.83 (5.87, 17.79); p ≤ 0.001	8.66 (3.72, 13.61); p ≤ 0.001	2.46 (1.53, 3.97); p ≤ 0.001
Q17: Room temperature vs Refrigerator	568	-4.09 (-24.67, 16.49); p = 0.697	-5.38 (-24.04, 13.27); p = 0.571	1.17 (0.31, 4.32); p = 0.819
Q18: Always vs Less than always	571	0.49 (-3.85, 4.83); p = 0.824	3.05 (-0.54, 6.63); p = 0.095	1.36 (0.95, 1.93); p = 0.090
Q19: Twice/multiple vs Only once	571	-8.81 (-15.43, -2.19); p = 0.009	-8.80 (-14.38, -3.23); p = 0.002	0.56 (0.34, 0.92); p = 0.023
Q19: Unsure vs Only once	571	-4.34 (-9.80, 1.12); p = 0.119	-6.62 (-11.09, -2.16); p = 0.004	0.58 (0.37, 0.89); p = 0.013
Q20: Cook then freeze leftovers vs Cook immediately	571	-11.73 (-19.30, -4.15); p = 0.002	-11.90 (-18.51, -5.29); p ≤ 0.001	0.24 (0.12, 0.52); p ≤ 0.001
Q20: Refreeze uncooked vs Cook immediately	571	-10.24 (-21.99, 1.51); p = 0.087	-10.44 (-20.37, -0.50); p = 0.040	0.47 (0.21, 1.06); p = 0.068
Q22: Smell/texture vs Juices	571	-4.16 (-8.90, 0.58); p = 0.085	-3.99 (-7.92, -0.06); p = 0.046	0.78 (0.53, 1.15); p = 0.213
Q22: Thermometer vs Juices	571	-11.97 (-20.26, -3.68); p = 0.005	-4.67 (-11.50, 2.16); p = 0.180	1.07 (0.60, 1.92); p = 0.816
Q22: No check vs Juices	571	-26.53 (-41.74, -11.33); p ≤ 0.001	-21.74 (-34.39, -9.09); p ≤ 0.001	0.24 (0.08, 0.74); p = 0.013
Q23: After 30 minutes vs Within 30 minutes	571	-7.67 (-13.82, -1.52); p = 0.015	-7.60 (-12.73, -2.47); p = 0.004	0.64 (0.41, 1.01); p = 0.056
Q24: Microwave vs Stove/oven	571	-6.06 (-10.64, -1.48); p = 0.010	-2.83 (-6.56, 0.90); p = 0.136	0.72 (0.49, 1.06); p = 0.097
Q24: No reheating/cold vs Stove/oven	571	-11.33 (-19.34, -3.32); p = 0.006	-7.07 (-13.84, -0.30); p = 0.041	0.56 (0.31, 1.00); p = 0.049

Table S2. Practice/belief associations: Contradictory question scored zero

Each row comes from a separate question model adjusted for age, sex, education, occupation, and region. Entries give estimate (95% CI); p. All fitted contrasts are shown. Continuous-score estimates are percentage-point differences; binary estimates are ORs. Full demographic coefficients for every model are supplied in the analysis archive.

Contrast	N	Reduced 9 difference	Original 14 difference	Original threshold OR
Q14: Ever vs Never	598	-3.18 (-8.19, 1.83); p = 0.213	-1.89 (-6.08, 2.30); p = 0.376	Not fitted
Q15: Always vs Not always	598	5.39 (-0.42, 11.20); p = 0.069	7.83 (2.97, 12.69); p = 0.002	Not fitted
Q16: Always vs Not always	598	14.70 (6.41, 22.98); p ≤ 0.001	13.87 (6.96, 20.78); p ≤ 0.001	Not fitted
Q17: Freezer vs Refrigerator	595	10.19 (4.26, 16.13); p ≤ 0.001	7.15 (2.27, 12.04); p = 0.004	Not fitted
Q17: Room temperature vs Refrigerator	595	-4.57 (-23.90, 14.75); p = 0.642	-6.23 (-23.89, 11.43); p = 0.488	Not fitted
Q18: Always vs Less than always	598	-0.42 (-4.71, 3.88); p = 0.849	2.32 (-1.20, 5.84); p = 0.196	Not fitted
Q19: Twice/multiple vs Only once	598	-9.27 (-15.74, -2.80); p = 0.005	-8.90 (-14.30, -3.51); p = 0.001	Not fitted
Q19: Unsure vs Only once	598	-5.72 (-11.14, -0.29); p = 0.039	-7.63 (-12.06, -3.20); p ≤ 0.001	Not fitted
Q20: Cook then freeze leftovers vs Cook immediately	598	-11.59 (-18.94, -4.25); p = 0.002	-11.19 (-17.52, -4.87); p ≤ 0.001	Not fitted
Q20: Refreeze uncooked vs Cook immediately	598	-12.09 (-23.69, -0.49); p = 0.041	-11.50 (-21.23, -1.77); p = 0.021	Not fitted
Q22: Smell/texture vs Juices	598	-5.12 (-9.81, -0.42); p = 0.033	-4.74 (-8.60, -0.88); p = 0.016	Not fitted
Q22: Thermometer vs Juices	598	-11.58 (-19.65, -3.50); p = 0.005	-4.56 (-11.18, 2.05); p = 0.176	Not fitted
Q22: No check vs Juices	598	-26.77 (-40.68, -12.86); p ≤ 0.001	-21.93 (-33.07, -10.78); p ≤ 0.001	Not fitted
Q23: After 30 minutes vs Within 30 minutes	598	-6.99 (-13.13, -0.86); p = 0.026	-6.97 (-12.09, -1.86); p = 0.008	Not fitted
Q24: Microwave vs Stove/oven	598	-5.72 (-10.29, -1.15); p = 0.014	-2.70 (-6.40, 1.00); p = 0.152	Not fitted
Q24: No reheating/cold vs Stove/oven	598	-10.58 (-18.24, -2.93); p = 0.007	-6.69 (-13.13, -0.24); p = 0.042	Not fitted

Table S3. Bootstrap internal validation of full-sample Firth models

500 ordinary case-bootstrap resamples per model, seed 20260831. Coding and adjustment terms remain fixed; the model is refitted in each resample. Corrected performance = apparent performance minus mean training-minus-test optimism. Test data are the original analysis sample. All 500 fits succeeded in each model. These diagnostics apply to logistic sensitivity models, not to instrument validity or causal effects.

Model	N	Metric	Apparent	Mean optimism	Corrected
Demographics	598	AUC	0.646	0.044	0.602
Demographics	598	Brier	0.232	-0.012	0.245
Demographics	598	Calibration slope	1.038	0.314	0.724
Q14	598	AUC	0.645	0.047	0.598
Q14	598	Brier	0.232	-0.013	0.245
Q14	598	Calibration slope	1.040	0.325	0.716
Q15	598	AUC	0.652	0.045	0.607
Q15	598	Brier	0.231	-0.013	0.244
Q15	598	Calibration slope	1.040	0.313	0.727
Q16	598	AUC	0.669	0.042	0.627
Q16	598	Brier	0.228	-0.013	0.241
Q16	598	Calibration slope	1.039	0.281	0.759
Q17	595	AUC	0.671	0.042	0.629
Q17	595	Brier	0.226	-0.013	0.240
Q17	595	Calibration slope	1.041	0.276	0.765
Q18	598	AUC	0.644	0.046	0.598
Q18	598	Brier	0.232	-0.013	0.245
Q18	598	Calibration slope	1.040	0.323	0.717
Q19	598	AUC	0.660	0.044	0.615
Q19	598	Brier	0.228	-0.014	0.242
Q19	598	Calibration slope	1.039	0.291	0.748
Q20	598	AUC	0.679	0.042	0.637
Q20	598	Brier	0.225	-0.013	0.238
Q20	598	Calibration slope	1.044	0.272	0.772
Q22	598	AUC	0.661	0.049	0.612
Q22	598	Brier	0.230	-0.014	0.244
Q22	598	Calibration slope	1.045	0.331	0.713
Q23	598	AUC	0.654	0.045	0.608
Q23	598	Brier	0.231	-0.013	0.244
Q23	598	Calibration slope	1.041	0.318	0.722
Q24	598	AUC	0.655	0.048	0.607
Q24	598	Brier	0.231	-0.014	0.244
Q24	598	Calibration slope	1.042	0.323	0.719

Table S4. Analysis populations and category definitions

Population	N	Interpretation
Full complete-case sample	598	624 records; 3 missing age, then 23 additional incomplete records excluded
Meat consumers	580	Q6 above zero
Exclude explicit non-responsibility	339	Remove 259 selecting the relevant Q25 barrier; remaining respondents are not confirmed preparers
Intersection	331	Meat consumers who did not select explicit non-responsibility
Exclude inconsistent responses	571	Remove 27 distinct respondents with keyed and do-not-know selections

Inconsistent responses: Q8 = 6, Q9 = 10, Q13 = 16; their union is 27. The zero-scoring sensitivity retains 598 respondents and assigns zero to the entire affected scored question. It does not retrospectively change the original stored responses.

Variable	Revised categories (first is reference)
Age	18-24; 25-34; 35-44; 45-55; >55
Sex	Male; Female
Education	High school or less; Diploma; Bachelor; Postgraduate
Occupation	Student; Employed/self-employed; Unemployed; Retired
Region	Eastern; Central; Western; Northern; Southern
Q14	Never; Ever
Q15	Not always; Always
Q16	Not always; Always
Q17	Refrigerator; Freezer; Room temperature
Q18	Less than always; Always
Q19	Only once; Twice/multiple; Unsure
Q20	Cook immediately; Cook then freeze leftovers; Refreeze uncooked
Q22	Juices; Smell/texture; Thermometer; No check
Q23	Within 30 minutes; After 30 minutes
Q24	Stove/oven; Microwave; No reheating/cold

Q17 excludes other/no storage only for storage models. No unsafe/safe designation is assigned to the pooled categories. Education pools below-high-school with high-school; occupation pools employed with self-employed. Full coefficients and exact model denominators are in model_results.csv.

Table S5. Original practice responses by applicability subgroup

Each entry is n/N (%). Full = 598; meat consumers = 580; exclusion of explicit non-responsibility = 339; intersection = 331. These are respondent denominators, including multiple-response Q21. No column establishes confirmed food-preparation involvement.

Question	Original response	Full	Consumers	Exclude non-responsibility	Intersection
Item_14	Never – I do not consume raw or undercooked meat.	436/598 (72.9%)	421/580 (72.6%)	235/339 (69.3%)	229/331 (69.2%)
Item_14	Rarely – I have tried it once or a few times.	72/598 (12.0%)	71/580 (12.2%)	45/339 (13.3%)	45/331 (13.6%)
Item_14	Occasionally – I eat it from time to time.	72/598 (12.0%)	71/580 (12.2%)	45/339 (13.3%)	44/331 (13.3%)
Item_14	Frequently – I consume raw or undercooked meat regularly.	18/598 (3.0%)	17/580 (2.9%)	14/339 (4.1%)	13/331 (3.9%)
Item_15	Never – I wasn't aware it was necessary.	22/598 (3.7%)	20/580 (3.4%)	10/339 (2.9%)	10/331 (3.0%)
Item_15	Rarely – I don't usually think about it.	28/598 (4.7%)	26/580 (4.5%)	18/339 (5.3%)	17/331 (5.1%)
Item_15	Sometimes – I wash my hands, but not every time.	78/598 (13.0%)	77/580 (13.3%)	56/339 (16.5%)	55/331 (16.6%)
Item_15	Always – I wash my hands every time before handling raw meat.	470/598 (78.6%)	457/580 (78.8%)	255/339 (75.2%)	249/331 (75.2%)
Item_16	Never – I wasn't aware it was necessary.	15/598 (2.5%)	13/580 (2.2%)	7/339 (2.1%)	7/331 (2.1%)
Item_16	Rarely – I usually forget or skip this step.	16/598 (2.7%)	16/580 (2.8%)	10/339 (2.9%)	10/331 (3.0%)
Item_16	Sometimes – I wash my hands, but not every time.	30/598 (5.0%)	30/580 (5.2%)	23/339 (6.8%)	23/331 (6.9%)
Item_16	Always – I wash my hands immediately after handling raw meat.	537/598 (89.8%)	521/580 (89.8%)	299/339 (88.2%)	291/331 (87.9%)
Item_17	In the refrigerator, separated from other foods.	93/598 (15.6%)	91/580 (15.7%)	56/339 (16.5%)	55/331 (16.6%)
Item_17	In the refrigerator, but stored together with other foods (not separated).	32/598 (5.4%)	30/580 (5.2%)	24/339 (7.1%)	22/331 (6.6%)
Item_17	In the freezer until ready to use.	457/598 (76.4%)	444/580 (76.6%)	248/339 (73.2%)	244/331 (73.7%)
Item_17	At room temperature (not refrigerated or frozen).	13/598 (2.2%)	12/580 (2.1%)	11/339 (3.2%)	10/331 (3.0%)
Item_17	Others	2/598 (0.3%)	2/580 (0.3%)	0/339 (0.0%)	0/331 (0.0%)
Item_17	I don't store raw meat at home.	1/598 (0.2%)	1/580 (0.2%)	0/339 (0.0%)	0/331 (0.0%)
Item_18	Never	39/598 (6.5%)	35/580 (6.0%)	17/339 (5.0%)	17/331 (5.1%)
Item_18	Rarely	36/598 (6.0%)	36/580 (6.2%)	17/339 (5.0%)	17/331 (5.1%)
Item_18	Sometimes	65/598 (10.9%)	64/580 (11.0%)	38/339 (11.2%)	38/331 (11.5%)
Item_18	Frequently	108/598 (18.1%)	105/580 (18.1%)	68/339 (20.1%)	66/331 (19.9%)
Item_18	Always	350/598 (58.5%)	340/580 (58.6%)	199/339 (58.7%)	193/331 (58.3%)
Item_19	Only once	362/598 (60.5%)	352/580 (60.7%)	215/339 (63.4%)	209/331 (63.1%)
Item_19	Twice	70/598 (11.7%)	70/580 (12.1%)	49/339 (14.5%)	49/331 (14.8%)
Item_19	Multiple times, as many as needed	28/598 (4.7%)	27/580 (4.7%)	17/339 (5.0%)	16/331 (4.8%)
Item_19	I don't know / Not sure	138/598 (23.1%)	131/580 (22.6%)	58/339 (17.1%)	57/331 (17.2%)
Item_20	Cook it immediately after thawing	510/598 (85.3%)	496/580 (85.5%)	282/339 (83.2%)	276/331 (83.4%)
Item_20	Cook it and then freeze leftovers again.	52/598 (8.7%)	51/580 (8.8%)	32/339 (9.4%)	32/331 (9.7%)
Item_20	Refreeze the meat without cooking it	23/598 (3.8%)	22/580 (3.8%)	18/339 (5.3%)	17/331 (5.1%)
Item_20	I thaw and refreeze meat multiple times without cooking	13/598 (2.2%)	11/580 (1.9%)	7/339 (2.1%)	6/331 (1.8%)

Question	Original response	Full	Consumers	Exclude non-responsibility	Intersection
Item_21	Bad or sour smell	501/598 (83.8%)	489/580 (84.3%)	277/339 (81.7%)	273/331 (82.5%)
Item_21	I do not know	39/598 (6.5%)	36/580 (6.2%)	15/339 (4.4%)	14/331 (4.2%)
Item_21	Unusual change in color	456/598 (76.3%)	446/580 (76.9%)	267/339 (78.8%)	262/331 (79.2%)
Item_21	Visible mold or slimy texture	417/598 (69.7%)	409/580 (70.5%)	220/339 (64.9%)	219/331 (66.2%)
Item_22	Cutting the meat to see if juices run clear	318/598 (53.2%)	312/580 (53.8%)	177/339 (52.2%)	174/331 (52.6%)
Item_22	Relying on smell or texture	190/598 (31.8%)	183/580 (31.6%)	104/339 (30.7%)	102/331 (30.8%)
Item_22	Using a food thermometer to check internal temperature	65/598 (10.9%)	63/580 (10.9%)	46/339 (13.6%)	45/331 (13.6%)
Item_22	I don't check if meat is thoroughly cooked	25/598 (4.2%)	22/580 (3.8%)	12/339 (3.5%)	10/331 (3.0%)
Item_23	Immediately	326/598 (54.5%)	316/580 (54.5%)	185/339 (54.6%)	182/331 (55.0%)
Item_23	Within 30 minutes	153/598 (25.6%)	149/580 (25.7%)	84/339 (24.8%)	82/331 (24.8%)
Item_23	Within 1 hour	72/598 (12.0%)	70/580 (12.1%)	44/339 (13.0%)	42/331 (12.7%)
Item_23	After more than 1 hour	47/598 (7.9%)	45/580 (7.8%)	26/339 (7.7%)	25/331 (7.6%)
Item_24	On the stove or oven	311/598 (52.0%)	303/580 (52.2%)	188/339 (55.5%)	185/331 (55.9%)
Item_24	Using a microwave	212/598 (35.5%)	207/580 (35.7%)	113/339 (33.3%)	110/331 (33.2%)
Item_24	I don't usually reheat leftover meat	58/598 (9.7%)	53/580 (9.1%)	25/339 (7.4%)	23/331 (6.9%)
Item_24	Eat it cold without reheating	17/598 (2.8%)	17/580 (2.9%)	13/339 (3.8%)	13/331 (3.9%)

Table S6. Practice/belief associations: Full

Each row comes from a separate question model adjusted for age, sex, education, occupation, and region. Entries give estimate (95% CI); p. All fitted contrasts are shown. Continuous-score estimates are percentage-point differences; binary estimates are ORs. Full demographic coefficients for every model are supplied in the analysis archive.

Contrast	N	Reduced 9 difference	Original 14 difference	Original threshold OR
Q14: Ever vs Never	598	-3.99 (-8.92, 0.93); p = 0.112	-2.71 (-6.84, 1.41); p = 0.197	0.81 (0.55, 1.20); p = 0.292
Q15: Always vs Not always	598	5.43 (-0.31, 11.16); p = 0.064	7.89 (3.05, 12.74); p = 0.001	1.46 (0.96, 2.21); p = 0.076
Q16: Always vs Not always	598	14.31 (6.05, 22.56); p ≤ 0.001	13.81 (6.94, 20.67); p ≤ 0.001	2.71 (1.47, 4.99); p = 0.001
Q17: Freezer vs Refrigerator	595	11.01 (5.16, 16.86); p ≤ 0.001	7.84 (2.99, 12.68); p = 0.002	2.33 (1.47, 3.70); p ≤ 0.001
Q17: Room temperature vs Refrigerator	595	-5.36 (-24.67, 13.95); p = 0.586	-6.46 (-23.97, 11.06); p = 0.469	1.02 (0.29, 3.62); p = 0.978
Q18: Always vs Less than always	598	-0.68 (-4.91, 3.55); p = 0.753	2.11 (-1.38, 5.60); p = 0.235	1.21 (0.86, 1.70); p = 0.282
Q19: Twice/multiple vs Only once	598	-9.24 (-15.56, -2.92); p = 0.004	-9.08 (-14.40, -3.77); p ≤ 0.001	0.53 (0.32, 0.86); p = 0.010
Q19: Unsure vs Only once	598	-5.29 (-10.66, 0.08); p = 0.053	-7.09 (-11.48, -2.70); p = 0.002	0.56 (0.37, 0.85); p = 0.007
Q20: Cook then freeze leftovers vs Cook immediately	598	-11.17 (-18.56, -3.78); p = 0.003	-11.20 (-17.61, -4.80); p ≤ 0.001	0.26 (0.13, 0.54); p ≤ 0.001
Q20: Refreeze uncooked vs Cook immediately	598	-9.65 (-20.87, 1.57); p = 0.092	-9.87 (-19.43, -0.30); p = 0.043	0.50 (0.23, 1.07); p = 0.074
Q22: Smell/texture vs Juices	598	-4.62 (-9.25, 0.01); p = 0.051	-4.30 (-8.11, -0.48); p = 0.027	0.78 (0.53, 1.14); p = 0.195
Q22: Thermometer vs Juices	598	-11.52 (-19.55, -3.49); p = 0.005	-4.56 (-11.15, 2.03); p = 0.175	1.07 (0.61, 1.88); p = 0.825
Q22: No check vs Juices	598	-19.40 (-34.27, -4.52); p = 0.011	-15.62 (-28.03, -3.21); p = 0.014	0.41 (0.16, 1.03); p = 0.059
Q23: After 30 minutes vs Within 30 minutes	598	-6.72 (-12.80, -0.64); p = 0.030	-6.82 (-11.92, -1.71); p = 0.009	0.71 (0.45, 1.10); p = 0.123
Q24: Microwave vs Stove/oven	598	-4.94 (-9.45, -0.44); p = 0.032	-2.07 (-5.73, 1.59); p = 0.268	0.79 (0.55, 1.15); p = 0.216
Q24: No reheating/cold vs Stove/oven	598	-10.89 (-18.48, -3.30); p = 0.005	-6.70 (-13.13, -0.27); p = 0.041	0.56 (0.32, 0.98); p = 0.043

Table S7. Practice/belief associations: Meat consumers

Each row comes from a separate question model adjusted for age, sex, education, occupation, and region. Entries give estimate (95% CI); p. All fitted contrasts are shown. Continuous-score estimates are percentage-point differences; binary estimates are ORs. Full demographic coefficients for every model are supplied in the analysis archive.

Contrast	N	Reduced 9 difference	Original 14 difference	Original threshold OR
Q14: Ever vs Never	580	-4.10 (-9.11, 0.90); p = 0.108	-2.57 (-6.76, 1.63); p = 0.230	0.83 (0.56, 1.22); p = 0.338
Q15: Always vs Not always	580	5.17 (-0.67, 11.01); p = 0.083	7.53 (2.61, 12.46); p = 0.003	1.43 (0.94, 2.18); p = 0.096
Q16: Always vs Not always	580	13.87 (5.61, 22.13); p = 0.001	13.08 (6.24, 19.93); p ≤ 0.001	2.61 (1.41, 4.82); p = 0.002
Q17: Freezer vs Refrigerator	577	10.87 (4.86, 16.88); p ≤ 0.001	7.78 (2.80, 12.76); p = 0.002	2.26 (1.42, 3.60); p ≤ 0.001
Q17: Room temperature vs Refrigerator	577	-5.96 (-27.01, 15.10); p = 0.579	-5.40 (-24.28, 13.49); p = 0.575	1.09 (0.30, 3.99); p = 0.894
Q18: Always vs Less than always	580	-0.43 (-4.69, 3.83); p = 0.844	2.14 (-1.39, 5.68); p = 0.234	1.22 (0.86, 1.73); p = 0.259
Q19: Twice/multiple vs Only once	580	-9.23 (-15.61, -2.85); p = 0.005	-8.78 (-14.14, -3.43); p = 0.001	0.54 (0.33, 0.87); p = 0.012
Q19: Unsure vs Only once	580	-4.43 (-9.85, 0.99); p = 0.109	-6.61 (-11.07, -2.16); p = 0.004	0.59 (0.39, 0.90); p = 0.015
Q20: Cook then freeze leftovers vs Cook immediately	580	-11.55 (-19.04, -4.07); p = 0.003	-11.55 (-18.06, -5.05); p ≤ 0.001	0.26 (0.13, 0.54); p ≤ 0.001
Q20: Refreeze uncooked vs Cook immediately	580	-6.26 (-17.50, 4.99); p = 0.275	-7.33 (-17.09, 2.42); p = 0.140	0.58 (0.27, 1.27); p = 0.173
Q22: Smell/texture vs Juices	580	-4.31 (-9.06, 0.43); p = 0.075	-4.01 (-7.92, -0.11); p = 0.044	0.81 (0.56, 1.20); p = 0.295
Q22: Thermometer vs Juices	580	-11.56 (-19.65, -3.47); p = 0.005	-4.90 (-11.61, 1.81); p = 0.152	1.07 (0.60, 1.90); p = 0.815
Q22: No check vs Juices	580	-16.18 (-32.21, -0.14); p = 0.048	-13.98 (-27.37, -0.58); p = 0.041	0.47 (0.18, 1.22); p = 0.120
Q23: After 30 minutes vs Within 30 minutes	580	-6.03 (-12.20, 0.15); p = 0.056	-6.14 (-11.32, -0.96); p = 0.020	0.73 (0.47, 1.15); p = 0.176
Q24: Microwave vs Stove/oven	580	-5.06 (-9.63, -0.49); p = 0.030	-2.08 (-5.81, 1.65); p = 0.275	0.79 (0.55, 1.15); p = 0.228
Q24: No reheating/cold vs Stove/oven	580	-10.02 (-17.84, -2.21); p = 0.012	-6.21 (-12.84, 0.42); p = 0.066	0.55 (0.31, 0.99); p = 0.046

Table S8. Practice/belief associations: Exclude explicit non-responsibility

Each row comes from a separate question model adjusted for age, sex, education, occupation, and region. Entries give estimate (95% CI); p. All fitted contrasts are shown. Continuous-score estimates are percentage-point differences; binary estimates are ORs. Full demographic coefficients for every model are supplied in the analysis archive.

Contrast	N	Reduced 9 difference	Original 14 difference	Original threshold OR
Q14: Ever vs Never	339	-3.25 (-10.17, 3.67); p = 0.357	-2.10 (-7.88, 3.68); p = 0.475	0.89 (0.53, 1.51); p = 0.673
Q15: Always vs Not always	339	4.83 (-2.16, 11.81); p = 0.175	7.57 (1.77, 13.37); p = 0.011	1.43 (0.83, 2.46); p = 0.199
Q16: Always vs Not always	339	12.33 (1.84, 22.82); p = 0.021	11.39 (2.83, 19.95); p = 0.009	2.19 (1.01, 4.77); p = 0.047
Q17: Freezer vs Refrigerator	339	12.46 (4.58, 20.33); p = 0.002	9.96 (3.53, 16.40); p = 0.003	2.55 (1.33, 4.89); p = 0.005
Q17: Room temperature vs Refrigerator	339	3.43 (-15.58, 22.45); p = 0.723	2.16 (-14.69, 19.02); p = 0.801	1.73 (0.43, 6.99); p = 0.439
Q18: Always vs Less than always	339	-4.03 (-9.90, 1.84); p = 0.178	-0.52 (-5.31, 4.26); p = 0.829	1.08 (0.67, 1.72); p = 0.760
Q19: Twice/multiple vs Only once	339	-7.28 (-15.82, 1.25); p = 0.094	-7.59 (-14.83, -0.35); p = 0.040	0.74 (0.40, 1.37); p = 0.337
Q19: Unsure vs Only once	339	-5.58 (-14.03, 2.87); p = 0.195	-6.95 (-13.93, 0.02); p = 0.051	0.52 (0.27, 1.02); p = 0.056
Q20: Cook then freeze leftovers vs Cook immediately	339	-7.98 (-18.01, 2.04); p = 0.118	-9.71 (-17.88, -1.54); p = 0.020	0.34 (0.13, 0.86); p = 0.022
Q20: Refreeze uncooked vs Cook immediately	339	-8.97 (-22.30, 4.36); p = 0.186	-9.33 (-20.55, 1.89); p = 0.103	0.57 (0.21, 1.51); p = 0.260
Q22: Smell/texture vs Juices	339	-4.63 (-11.04, 1.79); p = 0.157	-4.15 (-9.47, 1.18); p = 0.127	0.84 (0.49, 1.43); p = 0.520
Q22: Thermometer vs Juices	339	-14.85 (-24.63, -5.08); p = 0.003	-6.43 (-14.46, 1.60); p = 0.116	0.82 (0.40, 1.69); p = 0.597
Q22: No check vs Juices	339	-24.21 (-43.99, -4.44); p = 0.017	-17.71 (-32.92, -2.51); p = 0.023	0.26 (0.06, 1.16); p = 0.076
Q23: After 30 minutes vs Within 30 minutes	339	-11.85 (-19.77, -3.92); p = 0.004	-11.42 (-17.83, -5.00); p ≤ 0.001	0.44 (0.23, 0.84); p = 0.013
Q24: Microwave vs Stove/oven	339	-5.35 (-12.30, 1.61); p = 0.131	0.06 (-5.55, 5.67); p = 0.983	0.90 (0.54, 1.52); p = 0.704
Q24: No reheating/cold vs Stove/oven	339	-8.30 (-16.96, 0.36); p = 0.060	-3.88 (-11.22, 3.45); p = 0.298	0.35 (0.14, 0.88); p = 0.026

Table S9. Practice/belief associations: Intersection

Each row comes from a separate question model adjusted for age, sex, education, occupation, and region. Entries give estimate (95% CI); p. All fitted contrasts are shown. Continuous-score estimates are percentage-point differences; binary estimates are ORs. Full demographic coefficients for every model are supplied in the analysis archive.

Contrast	N	Reduced 9 difference	Original 14 difference	Original threshold OR
Q14: Ever vs Never	331	-3.63 (-10.61, 3.36); p = 0.308	-2.09 (-7.96, 3.77); p = 0.483	0.90 (0.53, 1.52); p = 0.691
Q15: Always vs Not always	331	5.27 (-1.86, 12.40); p = 0.147	7.58 (1.66, 13.51); p = 0.012	1.42 (0.82, 2.45); p = 0.208
Q16: Always vs Not always	331	13.02 (2.55, 23.49); p = 0.015	11.68 (3.14, 20.22); p = 0.007	2.24 (1.03, 4.86); p = 0.041
Q17: Freezer vs Refrigerator	331	12.62 (4.60, 20.65); p = 0.002	9.99 (3.38, 16.59); p = 0.003	2.47 (1.29, 4.75); p = 0.007
Q17: Room temperature vs Refrigerator	331	3.22 (-17.80, 24.25); p = 0.763	4.04 (-13.82, 21.90); p = 0.656	1.90 (0.45, 7.99); p = 0.380
Q18: Always vs Less than always	331	-3.59 (-9.51, 2.32); p = 0.233	-0.56 (-5.43, 4.32); p = 0.822	1.08 (0.67, 1.73); p = 0.748
Q19: Twice/multiple vs Only once	331	-7.88 (-16.56, 0.80); p = 0.075	-7.55 (-14.89, -0.21); p = 0.044	0.74 (0.39, 1.37); p = 0.333
Q19: Unsure vs Only once	331	-5.19 (-13.50, 3.12); p = 0.220	-7.02 (-14.07, 0.03); p = 0.051	0.53 (0.27, 1.03); p = 0.060
Q20: Cook then freeze leftovers vs Cook immediately	331	-8.71 (-18.72, 1.30); p = 0.088	-9.95 (-18.16, -1.73); p = 0.018	0.33 (0.13, 0.84); p = 0.020
Q20: Refreeze uncooked vs Cook immediately	331	-7.11 (-21.01, 6.79); p = 0.315	-8.26 (-20.30, 3.78); p = 0.178	0.65 (0.24, 1.77); p = 0.401
Q22: Smell/texture vs Juices	331	-4.79 (-11.32, 1.75); p = 0.151	-4.14 (-9.56, 1.28); p = 0.134	0.85 (0.49, 1.45); p = 0.545
Q22: Thermometer vs Juices	331	-14.39 (-24.26, -4.52); p = 0.004	-6.45 (-14.63, 1.72); p = 0.121	0.84 (0.41, 1.73); p = 0.641
Q22: No check vs Juices	331	-23.35 (-47.09, 0.38); p = 0.054	-19.65 (-37.73, -1.57); p = 0.033	0.28 (0.06, 1.34); p = 0.112
Q23: After 30 minutes vs Within 30 minutes	331	-12.09 (-20.33, -3.84); p = 0.004	-11.59 (-18.25, -4.93); p ≤ 0.001	0.45 (0.24, 0.87); p = 0.017
Q24: Microwave vs Stove/oven	331	-5.08 (-12.15, 2.00); p = 0.159	0.16 (-5.59, 5.90); p = 0.957	0.91 (0.54, 1.54); p = 0.734
Q24: No reheating/cold vs Stove/oven	331	-7.86 (-16.83, 1.11); p = 0.086	-3.76 (-11.52, 3.99); p = 0.340	0.32 (0.12, 0.84); p = 0.020

Supplementary File S1. Questionnaire English Version of the Questionnaire Used to Assess Public Knowledge and Practices Related to Bacterial Food Poisoning Associated with Meat Consumption in Saudi Arabia

Section 1. Demographic Information

1. Please indicate your age group:

- 18–24 years
- 25–34 years
- 35–44 years
- 45–55 years
- Older than 55 years

2. Sex:

- Male
- Female

3. What is the highest level of education you have completed?

- Postgraduate degree (Master's/PhD)
- Bachelor's degree
- Diploma/technical certificate
- High school
- Less than high school

4. Occupation:

- Student
- Employed
- Self-employed
- Unemployed
- Retired

5. In which region of Saudi Arabia do you currently reside?

- Central Region
- Eastern Region

- Western Region
- Northern Region
- Southern Region

6. Which of the following best describes your meat consumption?

- High meat consumption, approximately 75% of my meals or more
- Moderate meat consumption, approximately 50% of my meals
- Modest meat consumption, approximately 25% of my meals or less
- I do not consume meat (vegetarian)

Section 2. Knowledge of Bacterial Food Poisoning

7. Are you aware that food poisoning related to meat consumption may occur as a result of bacterial contamination?

- Yes, I am fully aware of this
- I have heard about it, but I do not know the details
- No, I was not aware of this
- I am not sure

8. In your opinion, which of the following practices may cause bacterial food poisoning related to meat consumption? (Select all that apply)

- Consuming raw or undercooked meat
- Poor hand hygiene
- Cross-contamination during meat preparation
- Improper meat storage
- I do not know
- Other

9. In your opinion, which of the following are common symptoms associated with bacterial food poisoning caused by contaminated meat? (Select all that apply)

- Nausea
- Vomiting
- Diarrhea
- Abdominal pain

- Fever
- I do not know

10. Are you aware of specific treatments used to manage bacterial food poisoning caused by contaminated meat?

- Yes, I am aware of specific treatments
- I have a general idea but do not know the details
- No, I am not aware of any specific treatments
- I am not sure

11. What do you usually do when you or a family member develops signs of food poisoning? (Select all that apply)

- Self-medicate using over-the-counter medications
- Consult a healthcare provider
- Use home remedies (e.g., herbal drinks, yogurt)
- Go to the hospital
- Wait for the symptoms to resolve without intervention
- I do not know what to do

12. Where have you obtained most of your information about meat handling and meat safety? (Select all that apply)

- School or university courses
- Social media platforms
- Television or radio
- Family or friends
- Personal experience
- Healthcare professionals
- I have not received any education or information about food safety

13. Which of the following treatments do you think are appropriate for bacterial food poisoning related to meat consumption? (Select all that apply)

- Drinking fluids and oral rehydration solutions
- Using over-the-counter antidiarrheal medications (e.g., loperamide)

- Taking antibiotics prescribed by a physician
- Using home remedies (e.g., herbal tea, yogurt)
- I do not know

Section 3. Meat-Handling Practices: Storage, Cooking, and Consumption

14. How often do you consume raw or undercooked meat?

- Frequently — I regularly consume raw or undercooked meat
- Occasionally — I consume it from time to time
- Rarely — I have tried it only once or a few times
- Never — I do not consume raw or undercooked meat

15. How often do you wash your hands before handling raw meat?

- Always — I wash my hands every time before handling raw meat
- Sometimes — I wash my hands, but not every time
- Rarely — I do not usually think about it
- Never — I was not aware that this was necessary

16. How often do you wash your hands after handling raw meat?

- Always — I wash my hands immediately after handling raw meat
- Sometimes — I wash my hands, but not every time
- Rarely — I often forget or skip this step
- Never — I was not aware that this was necessary

17. How do you usually store raw meat at home?

- In the refrigerator, separated from other foods
- In the refrigerator, stored together with other foods without separation
- In the freezer until ready to use
- At room temperature, without refrigeration or freezing
- Other

18. How often do you use specific utensils, such as knives and cutting boards, for handling raw and cooked meat?

- Always

- Frequently
- Sometimes
- Rarely
- Never

19. How many times do you think it is safe to thaw and refreeze meat?

- Only once
- Twice
- Multiple times, as needed
- I do not know / I am not sure

20. After thawing meat, what do you usually do? Please select the option that best describes your usual practice.

- Cook it immediately after thawing
- Refreeze the meat without cooking it
- Cook it and then freeze the leftovers again
- Thaw and refreeze the meat several times without cooking it

21. How do you determine whether meat is unsafe for cooking? (Select all that apply)

- Unusual change in color
- Bad or sour smell
- Visible mold or a slimy texture
- I do not know

22. Which method do you use to check whether meat is thoroughly cooked?

- Use a food thermometer to check the internal temperature
- Cut the meat to check whether the juices run clear
- Rely on smell or texture
- I do not check whether the meat is thoroughly cooked

23. After cooking meat, how long do you usually wait before serving or eating it?

- Immediately
- Within 30 minutes

- Within 1 hour
- After more than 1 hour

24. How do you usually reheat leftover meat?

- Using a microwave
- On the stove or in the oven
- I eat it cold without reheating
- I do not usually reheat leftover meat

Section 4. Knowledge Gaps and Suggestions for Improvement

25. What difficulties prevent you from following recommended meat-safety practices? (Select all that apply)

- Lack of time
- Lack of knowledge or training
- Cost of appropriate tools or equipment (e.g., food thermometers or cutting boards)
- Cultural or traditional beliefs
- I do not consider food safety a priority
- I am not the person responsible for preparing food at home

26. Even when I know the correct practices for preventing food poisoning related to meat, I do not always follow them in my daily life.

- Strongly agree
- Agree
- Neutral
- Disagree
- Strongly disagree

27. In your opinion, how effective is the use of spices, such as garlic, black pepper, and cumin, in protecting meat from bacterial contamination?

- I believe they are very effective and help reduce bacterial contamination
- I believe they are partially effective and should be combined with other preservation methods
- I do not believe they have a clear effect on preserving meat
- I do not know / I have not heard of using spices for this purpose

28. In your opinion, would gaining more knowledge about meat safety help you improve your food-related behavior?

- Yes
- No
- I am not sure

29. In your opinion, can following meat-safety practices help prevent food poisoning related to meat consumption?

- Yes
- No
- I do not know

30. In your opinion, what are the best methods for increasing awareness of meat safety in Saudi Arabia? (Select all that apply)

- School-based programs
- Workshops or community events
- Social media campaigns (e.g., X/Twitter, Instagram, Snapchat)
- Television and radio advertisements
- Mobile applications or SMS alerts
- Government health-awareness campaigns
- Other
